# Unifying Passive Air Sampling with Paper Spray Ionization Mass Spectrometry for the Detection of Third-Hand Cigarette Smoke

**DOI:** 10.1021/jasms.6c00271

**Published:** 2026-09-08

**Authors:** Bo-Cheng Ke, Noor Hidayat Abu Bakar, Pawel L. Urban

**Affiliations:** Department of Chemistry, 34881 National Tsing Hua University, 101, Section 2, Kuang-Fu Rd., Hsinchu 300044, Taiwan

## Abstract

To mitigate human exposure to air pollutants amid the increasing chemicalization of daily life, simple and rapid monitoring methods are required. As individuals spend a significant portion of their time indoors, indoor air quality has become a critical factor affecting human health and is influenced by a wide range of indoor and outdoor pollution sources. The sorption and desorption of indoor air pollutants on solid surfaces (the so-called *sink effect*) can gradually increase pollutant concentrations and lead to changes in indoor air quality. In particular, third-hand smoke (THS), a persistent form of indoor pollution, poses potential health risks through inhalation, dermal contact, and ingestion. Air sampling and analysis can be tedious and costly. Here, we present a very simple method that unifies passive sampling of THS onto paper and subsequent analysis by paper spray ionization (PSI) mass spectrometry (MS). Mass spectrometric results revealed the presence of nicotine, a major constituent of tobacco smoke, in THS samples collected using a custom-built passive sampler, which was further confirmed by MS/MS analysis. The applicability of this approach was further extended to a forensic context by detecting nicotine on book pages, which were previously exposed to cigarette smoke, using filter papers as sampling media. Overall, passive air sampling onto paper—combined with PSI-MS—provides a simple, cost-effective, and time-efficient way for indoor air quality monitoring and forensic analysis.

## Graphical abstract

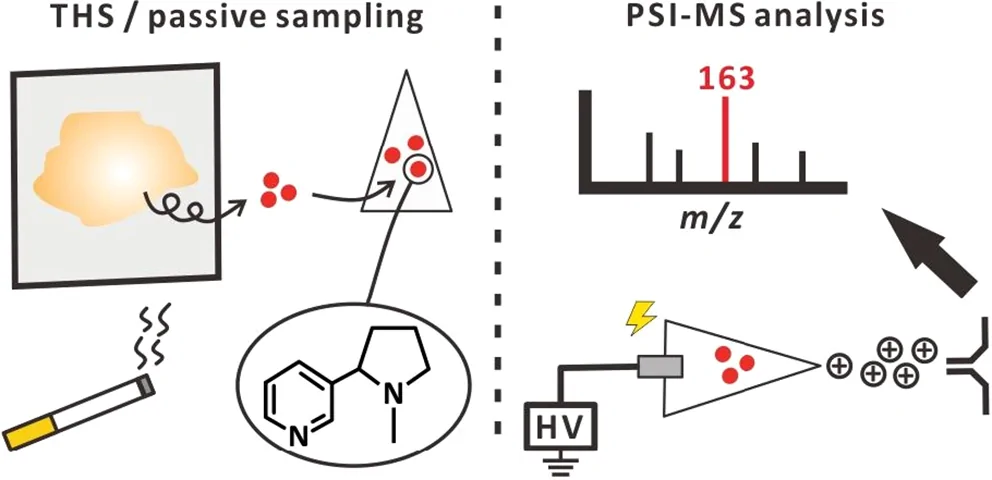

## Introduction

As people spend most of their lives indoors, indoor air quality (IAQ) has become a key determinant of human health, influenced by a variety of indoor and outdoor pollution sources.^1^ However, the sorption and desorption of indoor air pollution on solid material surfaces—a phenomenon known as the *sink effect*—can gradually increase pollutant concentrations and lead to changes in IAQ.^2^ Third-hand smoke (THS), which is the derivative of environmental tobacco smoke (ETS), represents a significant source of indoor pollution.^3^ THS is the residual tobacco-related contamination that persists on surfaces and within dust long after smoking has ceased, followed by the release of chemicals back into the air or the reaction with other compounds in the environment, forming carcinogenic tobacco-specific nitrosamines (TSNAs).^3,4^ Unlike THS, ETS—also referred to as secondhand smoke (SHS)—consists of mainstream smoke exhaled by smokers and sidestream smoke emitted from the burning tip of a cigarette.^5,6^ Nowadays, several toxic chemicals have been detected in THS, including nicotine, TSNAs, and polycyclic aromatic hydrocarbons (PAHs).^7^ While exposure to SHS occurs primarily through short-term inhalation, THS can lead to prolonged exposure through inhalation, dermal absorption, and ingestion, making it a distinct and persistent contributor to indoor air pollution, which poses threats to nonsmokers.^8^

Typically, monitoring of volatile organic compounds (VOCs) consists of sampling, pretreatment, separation, and detection.^9^ Air sampling represents one of the most critical and challenging steps in air pollution monitoring.^10^ In general, sampling techniques are classified into two categories: active (pumped) and passive (diffusive).^11^ Passive air sampling (PAS) operates via diffusive transport of analyte molecules from ambient air into a sorptive medium, eliminating the need for pumps or electrical power.^12^ Their simplicity, low cost, and maintenance-free design make them particularly advantageous for large-scale monitoring and for deployment in remote or resource-limited environments.^13^ Although PAS exhibits lower sampling rates compared to active methods, they provide time-integrated measurements that reflect average pollutant levels over extended periods.^14^ These characteristics make PAS an effective and practical tool for assessing long-term trends and spatial distributions of airborne contaminants, including those in indoor environments.^13^

To date, multiple techniques have been used to analyze THS, including GC-MS^3,15^ and LC-MS.^16^ However, the complicated sample preparation procedures of conventional methods make IAQ monitoring time-consuming. Therefore, there is a need to develop an approach capable of rapidly detecting THS with minimal sample pretreatment. Ambient ionization MS is a technique that enables direct analysis of raw samples in their native condition with little or no sample pretreatment.^17^ Commonly used techniques include desorption electrospray ionization (DESI), direct analysis in real time (DART), and paper spray ionization (PSI).^18^ In fact, one of the ambient ionization techniques—called desorption corona beam ionization (DCBI)^19^—was already applied for the detection of THS.^20^ PSI is a spray-based ambient ionization technique used for the rapid, quantitative, and qualitative analysis of complex mixtures. PSI consists of three major analytical steps, including sample collection, analyte separation, and analyte ionization.^21^ In brief, a small volume of sample is applied to a triangular paper substrate, such as chromatography paper and filter paper, followed by the addition of a spray solvent (typically a methanol/water mixture).^22^ The solvent migrates through the porous paper by capillary action, extracting analytes that are subsequently ionized when a high voltage (2–5.5 kV) is applied to the paper tip.^23^ Given its capability for rapid, direct analysis with minimal sample preparation, PSI has been applied to several fields, including forensic science^24^ and clinical diagnostics.^23,25^ However, PSI shows strong potential for environmental monitoring applications, particularly in air quality assessment, where it enables direct sampling and analysis of pollutants, such as tobacco smoke.^23,26^

In this study, we aim to establish a rapid approach for evaluating IAQ using PSI-MS. Specifically, this work integrates passive paper-based sampling with direct PSI-MS analysis to enable on-site detection of airborne pollutants without extensive sample preparation. The study focuses on one of the indoor contaminants—THS, which significantly affects indoor environments. It aims to develop a practical and adaptable platform for real-time and retrospective indoor air assessment, advancing the understanding of exposure risks associated with THS-related pollutants.

## Experimental Section

### Chemicals and Materials

Methanol (LC grade) was purchased from Merck (Darmstadt, Germany). Acetonitrile (LC-MS grade) and water (LC-MS grade) were purchased from Fisher Scientific (Waltham, MA). Formic acid (≥98%) was purchased from Thermo Fisher Scientific (Fair Lawn, NJ). (*S*)-(−)-Nicotine (99%) standard solution was purchased from Alfa Aesar (Ward Hill, MA). Pyrrolidine (>99%) was purchased from Acros Organics (Geel, Belgium). Quantitative ashless filter papers (No. 3; diameter, 90 mm) were purchased from Advantec (Tokyo, Japan). Cigarettes (with tar and nicotine levels of 8 mg per cigarette and 0.6 mg per cigarette, respectively, which are indicated on the pack) were purchased in a convenience store (Hsinchu, Taiwan).

### Preparation of Paper Substrates for Passive Sampling and Paper Spray Ionization Mass Spectrometric Analysis

Before air sampling and PSI-MS analysis, the paper substrates were prepared following a standardized procedure. Filter paper disks were first cut into isosceles triangular shapes (15 mm × 10 mm, height × base) using a razor blade. The cut pieces were sonicated in a 75% (v/v) aqueous methanol solution for 5 min, then replaced with fresh 75% (v/v) aqueous methanol and sonicated for another 5 min. Subsequently, the solvent was changed to pure acetonitrile, and the paper pieces were gently shaken (without sonication) for 5 min. Finally, the wetted paper pieces were dried by flushing with nitrogen gas for 30 min before use. To evaluate the effectiveness of the cleaning procedure, paper substrates before and after cleaning were analyzed by PSI-MS. As shown in Figure S1A, several ions with relatively high signal intensities (*e.g., m*/*z* 163, 205, and 222) were detected on the untreated paper. After the cleaning procedure, these signals were effectively eliminated (Figure S1B), demonstrating the effectiveness of the cleaning process in removing background contaminants.

### THS Simulation Chamber and Sampling Method

The simulation chamber (52 cm × 48 cm × 65 cm, L × W × H) was constructed from acrylic panels (thickness, 8 mm). A portable USB fan (XM66–28; Sheng De Fu Enterprise, Tainan, Taiwan) was placed inside to ensure uniform air mixing within the closed system. Passive sampler holders were designed to hold three triangular paper substrates (15 mm × 10 mm, height × base) for the collection of THS (Figure S2). They were fabricated by 3D printing (UP Plus 2; Beijing Tiertime Technology Company, Beijing, China) using ABS filament. 3D-printed materials may release contaminants,^27,28^ particularly when exposed to elevated temperatures, affecting the experiment. In the present study, however, the 3D-printed samplers were operated at room temperature, minimizing the potential for temperature-induced VOC emissions during sampling. Before introducing the THS source, the passive samplers were positioned in the chamber to verify the absence of residual tobacco smoke, which served as the “field blank”. To avoid interference from SHS, THS generation was conducted in two consecutive stages. In the first stage, SHS was generated by burning a cigarette for 8 min in a separate chamber, allowing the tobacco smoke to adsorb onto solid materials (*e.g*., paper towel and cotton cloth; 10 cm × 10 cm, L × W), as illustrated in Figure 1A (photo shown in Figure S3A). In the second stage, the smoke-laden substrates were transferred to the simulation chamber, where the adsorbed compounds were re-emitted and subsequently collected by passive samplers, as illustrated in Figure 1B (photo shown in Figure S3B). After 30 min of sampling, the samplers were removed from the chamber and directly subjected to PSI-MS analysis. Relative humidity (RH, %) and temperature (*T*, °C) were recorded at the time of sampler removal to monitor the experimental conditions for each sampling event.

**Figure 1:**
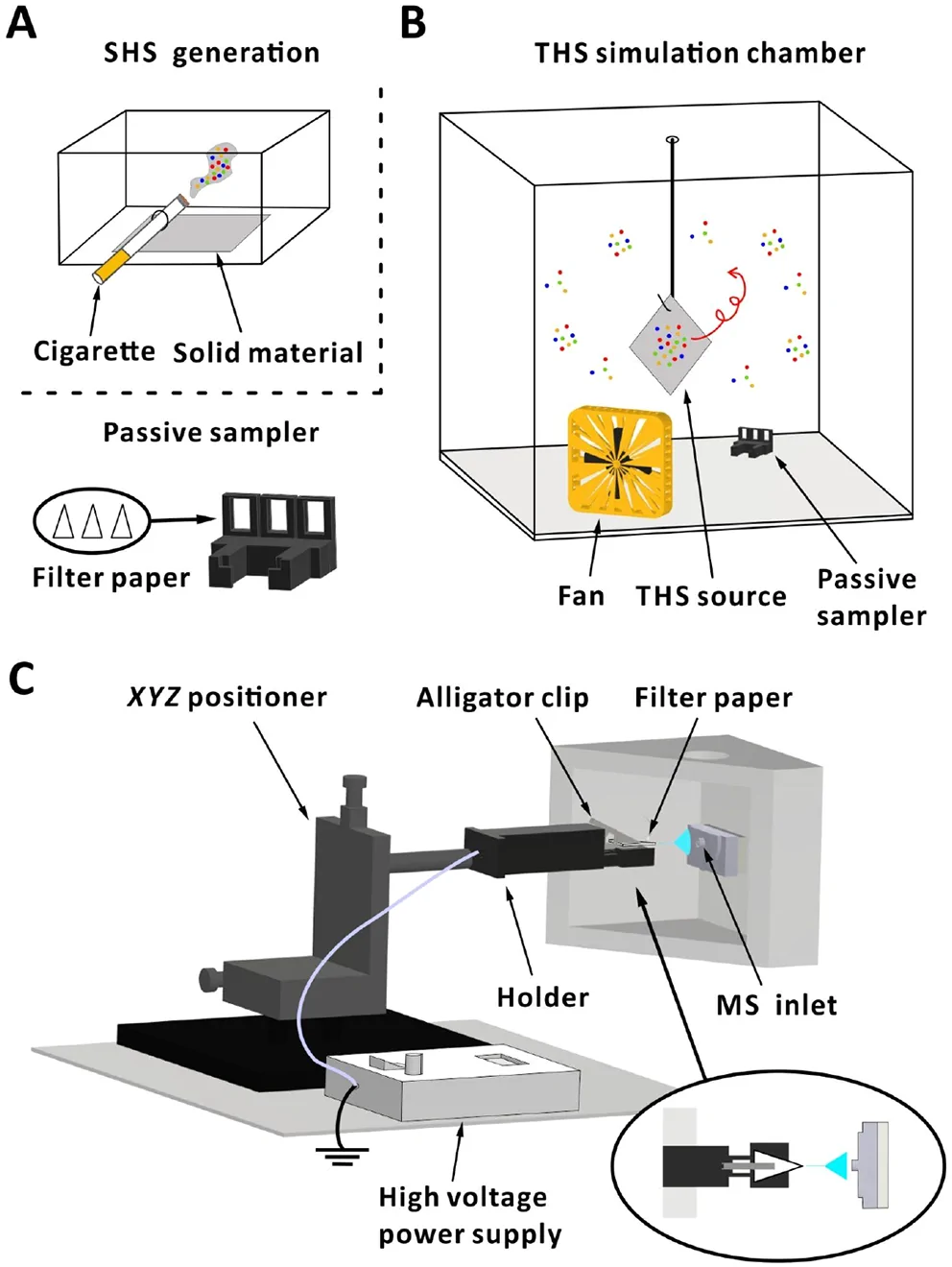
Experimental design: (A) production of SHS; (B) exposure of passive samplers to THS; (C) probe analysis by PSI-MS.

### Paper Spray Ionization Mass Spectrometric Analysis

For PSI-MS analysis, a triangular paper substrate was removed from the passive sampler and mounted onto the PSI device following the procedure described previously.^22^ As illustrated in Figure 1C (photo shown in Figure S3C), a custom-designed holder was introduced to support the paper substrates, preventing the paper tip from collapsing when the spray solvent was added, which otherwise could affect analytical stability. After air sampling, the prepared paper probes were mounted on the PSI device, positioned ∼10 mm away from the inlet of a triple quadrupole mass spectrometer (LCMS-8030; Shimadzu, Kyoto, Japan). Only the middle part of the filter paper has contact with the holder to minimize its effect on sample movement. The spray solvent consisted of a 20 μL aliquot of 75% (v/v) aqueous methanol containing 0.1% (v/v) formic acid. Pyrrolidine (20 μM)—used as an internal standard—was spiked into the spray solvent. The solvent was then applied to the rear portion of the paper using a pipet, allowing the solution to migrate through the paper via capillary action. Upon application of a +4.5 kV potential, charged droplets were generated from the paper tip, producing gas-phase ions that were subsequently analyzed by the mass spectrometer. Because PSI exhibits spray characteristics similar to those of nanoelectrospray ionization,^23^ drying gas was switched off during the PSI-MS analysis to avoid disruption of spray formation and to maintain signal stability^29,30^ (see also Figure S4). The heat block temperature was 400 °C while the desolvation line temperature was 250 °C and the desolvation line voltage was 0 V. The instrument was operated in the positive-ion full-scan mode across an *m*/*z* range of 20–500 with a total scan duration of 2 min. Product-ion scan mode (collision-induced dissociation with argon; collision energy: 20 to 30 V, varying across different compounds) was applied to further confirm the identities of MS signals.

## Results and Discussion

### Characterization of Direct Sampling and Analysis by PSI-MS

To evaluate the effectiveness of direct sampling and analysis by PSI-MS, the analytical performance of PSI-MS was compared with that of ESI-MS. PSI-MS represents a technique that requires no sample pretreatment, whereas extraction and sample preparation (dilution and addition of an acidic additive, 0.1% formic acid) were performed prior to ESI-MS analysis. As shown in Figure 2A, nicotine (*m*/*z* 163) was observed exclusively in the spectrum of the sample paper exposed to THS as a peak at *m*/*z* 163. Nicotine—being one of the most abundant components of tobacco smoke—plays a crucial role in THS chemistry due to its high emission rate, elevated surface concentrations, and persistence on indoor materials.^8,31,32^ Its identity was further confirmed by comparison of the tandem mass spectrum with that of a nicotine standard (Figure S5). The close agreement between the tandem mass spectra of nicotine obtained from THS (Figure S5C) and the nicotine standard (Figure S5D) indicates that THS constituents, particularly nicotine, were successfully collected by the passive samplers. Other tobacco-related compounds, including myosmine and nicotine 1′-*N*-oxide, were also detected in THS, and are discussed in the next section. In contrast, nicotine was not detected in the sampled paper analyzed by ESI-MS (Figure 2B). These results demonstrate that integrating direct sampling with PSI-MS analysis enables effective THS detection with minimal solvent consumption and without the need for sample pretreatment. The absence of the protonated nicotine peak (*m*/*z* 163) in the ESI mass spectrum may be attributed to several factors, with sample dilution likely being a major contributor. In PSI-MS analysis, only 20 μL of solvent were required for both extraction and ionization. To keep the sample preparation procedure as simple as possible and increase the sample volume to fill the syringe and ESI source flow line, ESI-MS analysis involved extraction with 1.5 mL of 75% (v/v) aqueous methanol through sonication, followed by an additional 2-fold dilution with the same solvent prior to MS analysis. This substantially greater dilution (150-fold) may have reduced the nicotine concentration to the point where it was below the detection limit. Moreover, PSI-MS enables direct analysis of compounds adsorbed onto the paper surface without sample pretreatment, allowing surface-bound residues to be ionized and detected under a high electric field. This direct ionization approach preserves the native chemical state of weakly adsorbed compounds, such as nicotine, allowing for rapid tobacco smoke analysis without the need for additional extraction or chromatographic separation.

**Figure 2:**
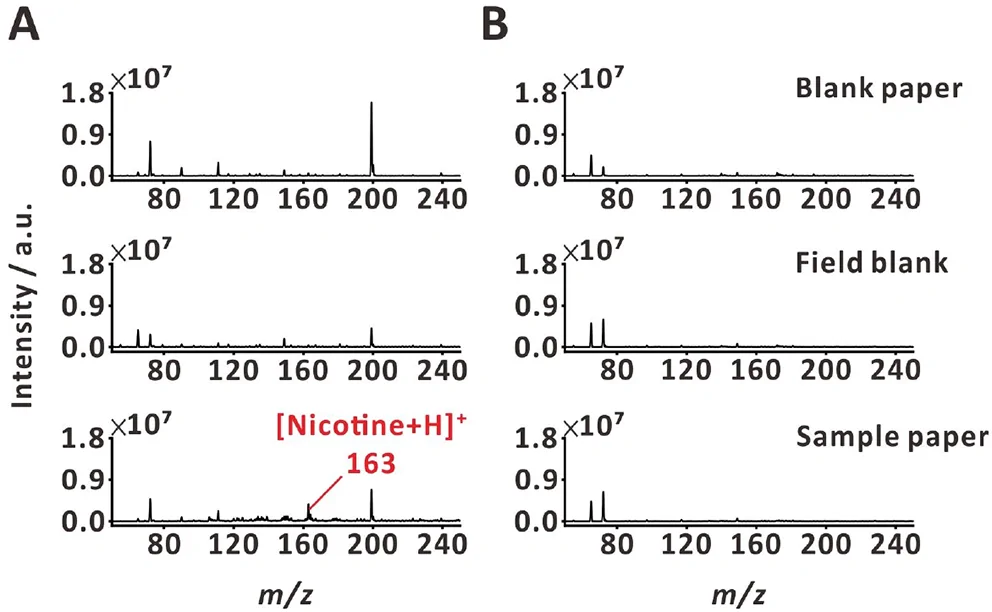
Mass spectra of THS collected from the test chamber and analyzed by (A) PSI-MS and (B) ESI-MS (RH: 58%; *T*: 17.6 °C).

Due to its abundance in tobacco smoke, nicotine was selected as the target compound for monitoring THS in the chamber experiment. To evaluate the analytical performance of this technique, a calibration curve was constructed using nicotine standards, with the structural analogue internal standard, pyrrolidine (*m*/*z* 72), spiked into the spray solvent. The limit of detection (LOD) and limit of quantitation (LOQ) were calculated using the following equations: LOD = 3.3σ/*S* and LOQ = 10σ/*S*, where σ represents the standard deviation of the *y*-intercepts of the linear regression, and *S* denotes the slope of the calibration curve. As shown in Figure S6, an LOD of 1.30 μM (210.90 ng mL^–1^) and an LOQ of 3.93 μM (637.56 ng mL^–1^) were obtained, with a linear range of 3–15 μM (*R* ^2^ = 0.9957). In addition, the relative standard deviation (RSD) was evaluated for both the nicotine standard and nicotine derived from THS. As shown in Figure S7A,B, the RSD for the nicotine standard was 13.5% (*n* = 10) when obtained for the nicotine-to-pyrrolidine signal ratios, compared to 24.9% when obtained from signal intensities, indicating improved precision with the use of an internal standard. This result is as expected because the internal standard partly compensates for signal drift during analysis. In addition, we investigated the repeatability of the THS samples by collecting air samples using 10 paper probes. As shown in Figure S7C, the RSD obtained for the nicotine-to-pyrrolidine ratios was 33.7% (*n* = 8). In this case, the RSD was calculated only for eight of the ten replicates due to the absence of the pyrrolidine signal (*m*/*z* 72) in replicates 3 and 10. Such signal loss may arise from slight but critical variations in substrate geometry during manual preparation. Besides, the RSD obtained from raw signal intensities was 51.6% (*n* = 10, Figure S7D). Although the RSD observed for real samples was higher than that obtained for the nicotine standard, this increased variability can be attributed to matrix effects and variations in the sampling process, reflecting the heterogeneous nature of THS and the inherent challenges of passive sampling. Such variability therefore provides an insight into the practical performance of the proposed approach under realistic sampling conditions. Overall, the proposed method can be regarded as a rapid qualitative or semiquantitative test for the presence of THS.

### SHS vs THS

THS refers to residual tobacco pollutants that accumulate on indoor surfaces and in dust, which can be re-emitted into the gas phase or undergo chemical transformations to form secondary pollutants.^4^ Meanwhile, SHS is a mixture of sidestream smoke emitted from burning tobacco products and mainstream smoke exhaled by smokers.^5,6^ In the next experiment, THS was generated following the procedures described above, whereas SHS was obtained by directly exposing passive samplers to sidestream tobacco smoke. The differences between SHS and THS were evaluated based on the mass spectra shown in Figure 3. SHS and THS consist of both VOCs and semivolatile organic compounds (SVOCs), including toluene, formaldehyde, nicotine, nitroamines, and PAHs.^4,7,33,34^ As shown in Figure 3, nicotine (structure shown in Figure S8D) was detected in both SHS and THS-derived paper samplers (*m*/*z* 163). Due to the unit mass resolution of the triple quadrupole mass spectrometer, peak assignment was further confirmed by MS/MS analysis. As shown in Figure S8A,B, the fragmentation patterns observed for SHS were similar to those for THS. According to Smyth and co-workers, the fragment ion at *m*/*z* 132 is due to the loss of CH_3_NH_2_ from the saturated heterocyclic ring, whereas the fragment at *m*/*z* 106 arises from the loss of C_3_H_5_NH_2_ (Figure S9).^35^ In addition, the assignment of the protonated nicotine peak was verified by comparison with the tandem mass spectrum of an authentic nicotine standard (Figure S8C). Notably, the signal intensity of nicotine in SHS was approximately four times higher than that in THS. This difference can be attributed to the direct collection of SHS from freshly emitted cigarette aerosols, whereas THS was derived from the off-gassing of volatile compounds re-emitted from contaminated surfaces. In addition to cellulose-based towel paper, cotton cloth—a commonly used textile material—was employed as another substrate to simulate THS formation. As shown in Figure S10, nicotine was detected in paper samplers exposed to THS emanating from both cotton cloth (Figure S10A) and towel paper (Figure S10B). Previous studies have reported nicotine sorption on common indoor materials, including cotton and glass.^36^ Although the sorption behavior of nicotine among different textile materials was not further compared in this study, these results demonstrate that cellulose-based materials can retain tobacco-related compounds and may serve as reservoirs for THS, potentially contributing to prolonged human exposure.

**Figure 3:**
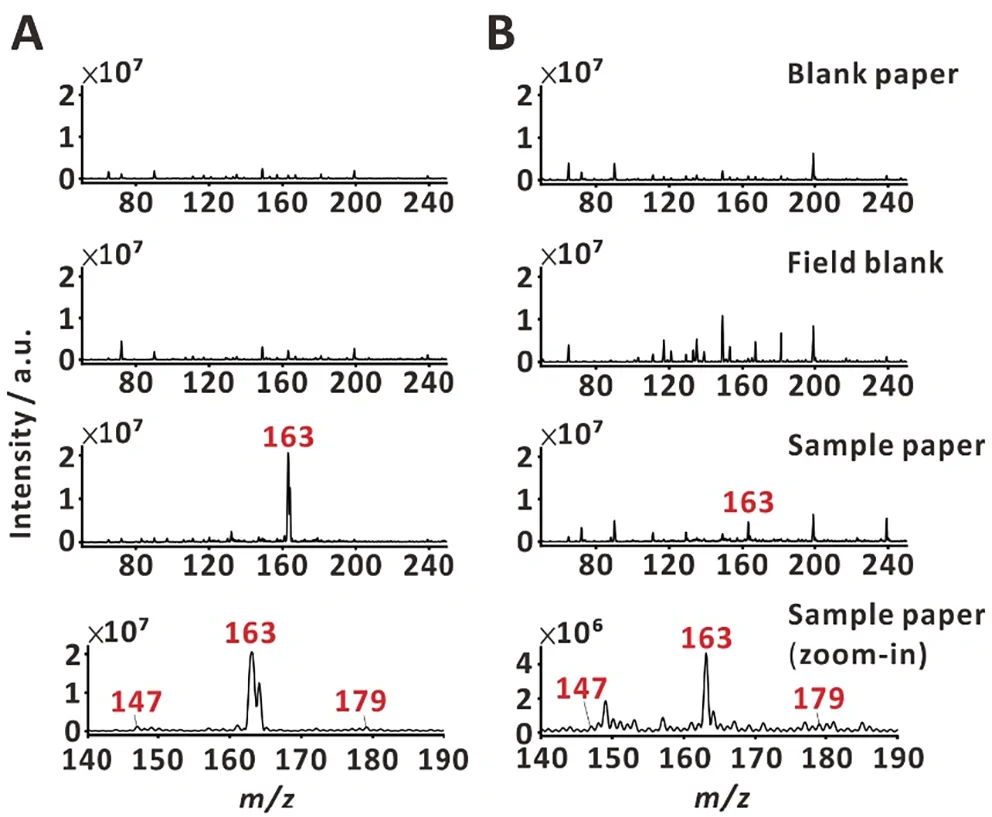
PSI-MS analysis of (A) SHS (RH: 81%; *T*: 21.6 °C) and (B) THS (RH: 78%; *T*: 21.5 °C). The zoom-in spectra shown in the last row indicate the peaks of compounds detected in the sample paper.

While THS contains lower concentrations of tobacco-related compounds than SHS, these residues can persist in indoor environments for extended periods and subsequently react with oxidants such as ozone and gaseous nitrous acid (HONO), leading to the formation of secondary organic aerosols (SOA) and TSNAs.^37,38^ Aside from nicotine, oxidation products of nicotine, including myosmine and nicotine 1′-*N*-oxide, were detected in both SHS and THS sampled by paper, with signal intensities more than an order of magnitude lower than that of nicotine (Figure 3). Myosmine (*m*/*z* 147; structure shown in Figure S11C), an oxidation product of nicotine and one of the earliest structurally characterized tobacco alkaloids aside from nicotine,^39^ was further confirmed by MS/MS analysis using the same approach as applied for nicotine. As shown in Figure S11, the fragmentation patterns observed in SHS (Figure S11A) closely resembled those in THS (Figure S11B), indicating that the same tobacco-related compound was present in both samples. According to the suggested fragmentations reported by Medana et al., the fragment ion at *m*/*z* 105 is attributed to the neutral loss of C_3_H_6_ (42 Da), while the ion at *m*/*z* 120 arises from the loss of HCN (27 Da; Figure S12).^40^ Nicotine 1′-*N*-oxide (*m*/*z* 179; structure shown in Figure S13C), a constituent of tobacco products, is generally considered to be formed via oxidative transformation of S-(−)-nicotine upon exposure to air during tobacco leaf processing, particularly during drying and curing.^41^ As shown in Figure S13A,B, the fragmentation patterns observed in SHS and THS were highly comparable, confirming the presence of nicotine 1′-*N*-oxide in both samples. The fragment ions at *m*/*z* 132 and 130 are consistent with the previously reported fragmentation pathways (Figure S14), including those described by Medana et al.^42^ and the tandem mass spectra reported by Uhlig et al.^43^

### Residual Smoke as a Function of Time Elapsed from the First Exposure

Indoor surfaces—such as furnishings and walls—can serve as sinks for VOCs, altering their concentrations through sorption and desorption (re-emission) processes, commonly referred to as the *sink effect*.^44^ In this study, the temporal variation of residual smoke concentration, particularly nicotine, was monitored over 300 min (5 h) in an acrylic test chamber (52 × 48 × 65 cm, L × W × H) at RH of 53 ± 1% and temperature of 23 ± 2 °C. Briefly, a cigarette was burned inside the chamber for 20 s and then removed. Monitoring was subsequently performed every hour, consisting of 30 min of equilibration followed by 30 min of sampling. The nicotine-to-pyrrolidine signal ratio was used to evaluate temporal changes in nicotine concentration. As shown in Figure 4, the nicotine/pyrrolidine ratio gradually decreased over time and approached a lower plateau. Error bars represent the standard deviation (*n* = 3), and the solid red line corresponds to a one-phase exponential decay fit. The exponential-like decay suggests that nicotine loss was dominated by a single apparent kinetic process within the investigated time range. Although nicotine re-emission was not directly observed within the experimental time frame, the progressive decrease in the detectable nicotine signal suggests time-dependent changes in the nicotine concentration present in the room. This observation is consistent with previous studies showing that nicotine, as a SVOC, readily partitions to indoor surfaces and can be slowly re-emitted over extended periods.^45,46^ While the chamber used in our test was made of acrylic rather than stainless steel, a temporal decay trend similar to that reported by Van Loy et al. was observed,^45^ suggesting that sink-related sorption behavior is not limited to metallic surfaces.

**Figure 4:**
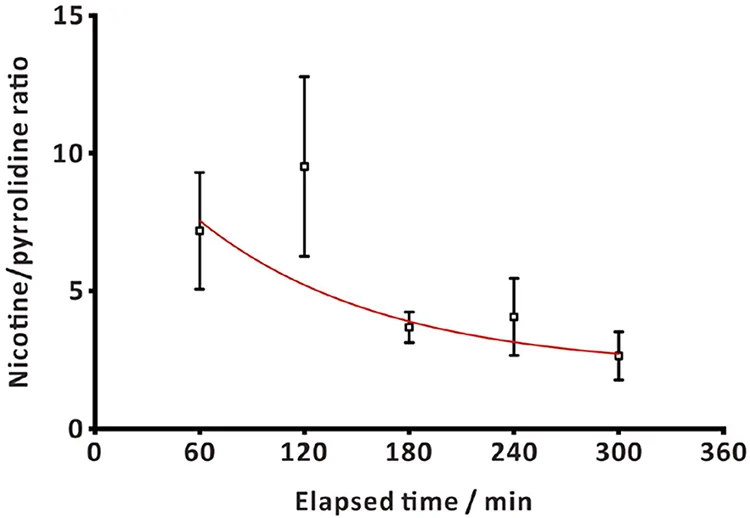
Temporal evolution of the nicotine-to-pyrrolidine ratio as a function of elapsed time (RH: 52–54%; *T*: 21.5–24.6 °C). Error bars represent mean ± standard deviation (*n* = 3) at each elapsed time, and the red curve represents an exponential decay fit.

Previous studies have demonstrated nicotine sorption on various indoor materials, including carpet, wallboard,^46^ glass, and textiles (*e.g*., cotton and nylon).^36^ Because acrylic is a polymeric material, nicotine retention may involve not only surface adsorption but also absorption and diffusion within the material matrix. Therefore, acrylic-based materials may act as long-term nicotine reservoirs that gradually re-emit THS-related compounds into indoor environments. These findings have important implications for indoor exposure and THS persistence, as acrylic and other plastic-based materials are widely used in offices, public spaces, furniture, and indoor partitions.

The observed time-dependent behavior is also influenced by environmental factors such as RH and temperature.^47^ Nicotine sorption depends on its protonation state and interfacial interactions, both of which can vary with environmental conditions.^48^ Ongwandee and Sawanyapanich reported that high RH (*e.g*., 90%) can enhance nicotine uptake by modifying adsorbed water layers and surface interaction sites.^49^ Temperature also affects both sorption equilibrium and mass transfer kinetics, thereby influencing the extent of nicotine retention on surfaces as well as its redistribution between phases.^50,51^ Collectively, these factors provide a mechanistic basis for the observed time-dependent behavior of THS, particularly nicotine, and highlight the importance of monitoring RH and temperature throughout the chamber experiments.

### Detection of Smoke Adsorbed on Pages of a Book

There have been anecdotal reports and complaints about THS being emitted from shared books; for example, in the library setting.^52^ One testimony mentioned: “(···) The books reeked of cigarette smoke. The odor was chokingly strong even from several feet away. (···)”^53^ Therefore, in another experiment—aimed to simulate a potential application of the developed method—an open book (teaching-related, 416 pages, purchased in Kaohsiung, Taiwan) was exposed to SHS from a burning cigarette for 10 min (Figure 5A). The smoke residues were primarily adsorbed on the surfaces of exposed pages but not on the other pages. Subsequently, triangle-shaped filter paper pieces were placed in between different pages of the same book (contact sampling; see Figure 5B), including the previously exposed (40 and 41), adjacent (38 and 39), and unexposed pages (10, 11, 20, and 21). The book was kept closed for 1 h, enabling direct contact of the paper samplers with the different book pages. The paper samplers were subsequently analyzed by PSI-MS to detect any traces of residual nicotine. Prior to tobacco smoke exposure, field blank analysis was conducted on the selected pages to ensure that no tobacco-related compounds were present in the unexposed book (Figure S15A). Tandem mass spectra shown in Figure S16A demonstrate very low amount of nicotine on unexposed book pages, as compared to the spectrum for the authentic standard (Figure S16C).

**Figure 5:**
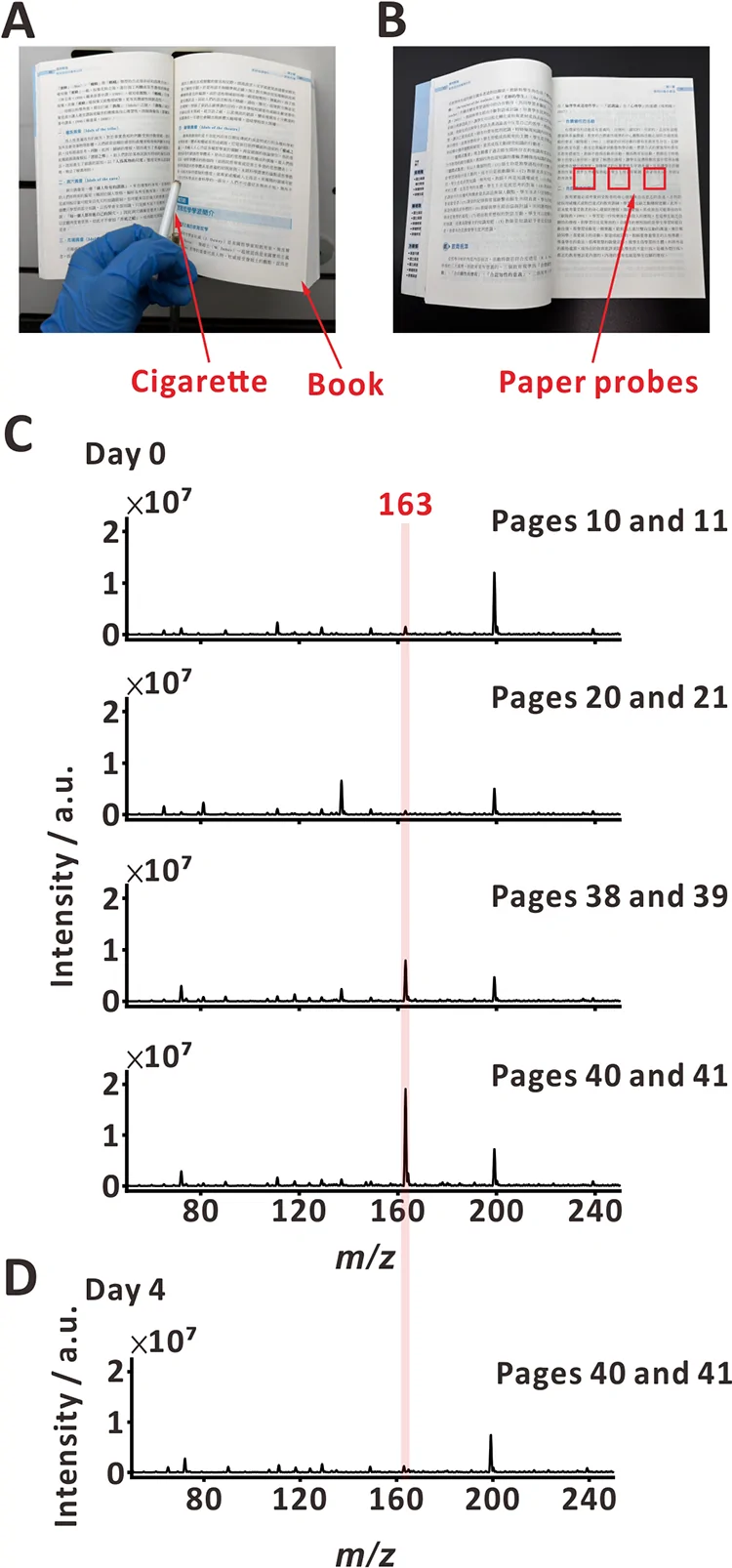
Illustration of THS sampling on book pages: (A) photo of tobacco smoke exposure to book pages (pages 40 and 41), (B) paper substrates placement on different pages (papers were labeled with a red square). Mass spectra of THS on book pages on day 0 (C) and after 4 days (D).

As shown in Figure 5C, nicotine (*m*/*z* 163) was detected on pages directly exposed to tobacco smoke (40 and 41), whereas lower signal intensities were observed on adjacent pages (38 and 39), and no detectable signal was found on more distant pages (10, 11, 20, and 21). This spatial distribution indicates that nicotine deposition under the present conditions was strongly dependent on proximity to the exposure source. The elevated signals on pages 40 and 41 are consistent with the efficient adsorption of tobacco smoke constituents onto solid surfaces. Nicotine is known to partition onto various indoor materials, including wool, polyester, and cellulose-based substrates, which can act as reservoirs.^54,55^ The detection of nicotine on unexposed adjacent pages suggests limited redistribution from the exposed region, likely through short-range transport within the closed book environment. In contrast, the absence of detectable nicotine on more distant pages indicates that transport into the interior of the book was minimal under the present experimental conditions.

To investigate temporal changes in nicotine levels, a second sampling was conducted 1 h after the initial measurement. As shown in Figure S15B, analysis of pages that previously showed no detectable nicotine (10, 11, 20, and 21) yielded values below the detection limit, whereas analysis of pages with detectable nicotine exhibited time-dependent nicotine signal changes. A pronounced decrease of nicotine signal was observed for the exposed pages and their adjacent pages, indicating that the amount of surface-accessible nicotine decreased over time. The detection of nicotine in book pages after exposure to smoke was further confirmed by tandem MS analysis, as shown in Figure S16B. This temporal evolution reflects the dynamic nature of nicotine–surface interactions, in which sorbed nicotine can undergo re-emission into the gas phase and subsequent redistribution within the indoor microenvironment.^56^ Mass transfer processes play a role in this behavior. Sorption is not limited to surface adsorption but also involves diffusion into the bulk of the material (in-material diffusion).^2,44^ As a typical SVOC, nicotine is expected to partition among the gas phase, surface, and bulk material rather than remaining solely on the outermost surface. Furthermore, adsorption on porous cellulose-based materials is governed by structural properties such as pore volume and surface area, indicating that nicotine can penetrate and be retained within internal pore networks as well as on surface sites.^57^ In addition to the variation in nicotine signal intensity across book pages, tobacco-related compounds such as myosmine and nicotine 1′-*N*-oxide—previously detected in SHS and THS—were not observed on the book pages.

To further evaluate the time-dependent evolution of nicotine signals over an extended interval, a third sampling was conducted on the same pages 4 days after the initial measurement. The book was kept closed during this period to minimize external contamination that could interfere with the detection of tobacco-related compounds. As shown in Figure 5D, nicotine remained detectable on the exposed pages after several days. Tandem mass spectra shown in Figure S17 reveal that nicotine was detected in the exposed pages (40 and 41) among all samplings after the tobacco smoke exposure, excluding the field blank. However, its signal intensity was approximately nine times lower than that observed in the initial (day 0) measurement. This observation is consistent with the previously discussed mechanism, in which diffusion of nicotine from surface layers into the internal structure of the material reduces the amount of surface-accessible nicotine, thereby diminishing the detectable signal over time.

To assess day-to-day reproducibility, the “book test” was performed repeatedly on three different days following the identical procedure described above. As shown in Figure S18 the nicotine-to-pyrrolidine ratio was used as a reproducible indicator for nicotine detection across different book pages, including both the initial sampling and the repeated sampling performed ∼1 h later. As expected, the measurements on the exposed pages (40 and 41; 110 and 111; 180 and 181) revealed the highest nicotine-to-pyrrolidine ratio among all the other pages. Moreover, the ratio decreased as the distance from the exposed pages increased. Adjacent pages (including 38 and 39; 108 and 109; 178 and 179) showed the second-highest ratio in all the measurements on the three different days, indicating that tobacco-related compounds—nicotine in this case— could also be detected on the unexposed pages close to the exposed pages. However, analysis of the unexposed pages—located at least ten pages away from the exposed pages—revealed comparable nicotine-to-pyrrolidine ratios to the field blank analysis among all the obtained values, indicating no nicotine detected in field blank, the initial THS samples, and the second samples. In addition, a sharp decrease in ratios was observed on both exposed and adjacent pages after the initial measurement, in agreement with the temporal decline discussed above. The consistent spatial distribution and temporal decay patterns—observed across three independent experiments—demonstrate satisfactory day-to-day reproducibility of this sampling and analytical approach, regardless of the specific page location examined.

## Conclusions

The surging chemicalization of daily life leads to increased exposure of humans to harmful substances calling for simple and efficient monitoring approaches. In the absence of active smokers, THS can be harmful to humans in home and office environments. Thus, IAQ monitoring focusing on THS is crucial for safety assurance. We have demonstrated a very simple method to detect the presence of cigarette smoke residues being emitted from common absorbers (paper, cloth). A novel aspect of this method is the use of paper for both passive THS sampling and MS analysis by PSI-MS. This integration simplifies and streamlines the analytical workflow reducing the sample handling steps in IAQ monitoring. Using the developed method, we observed sorption of nicotine from SHS into book pages and subsequent redistribution within the paper matrix. Thus, it has the potential applicability in sampling THS emitted by shared daily use objects such as books loaned from libraries. While it is often hard to point out the source of THS objectively, the proposed method addresses this issue to some extent. Moreover, the passive samplers can be attached to the elements of indoor infrastructure to collect traces of THS and evaluate the exposure of the indoor space users to harmful VOCs. To detect some highly abundant smoke components such as nicotine, a low-resolution mass spectrometer is sufficient. Therefore, the method can be used in the field in conjunction with portable mass spectrometers. Apart from detection of THS, the approach can potentially be utilized for IAQ monitoring in industrial facilities and osmology-based forensics.

## Supplementary Material



## Notes

Technical drawings and photographs of experimental setup; observations of the influence of drying gas on paper spray ionization; tandem mass spectra of tobacco-related compounds; calibration curve of nicotine by PSI-MS; repeatability test for nicotine standard and THS; suggest fragmentation pathway of tobacco-related compounds; emission of THS from different materials; mass spectra of book pages from blank analysis and second sampling; tandem mass spectra of precursor at *m/z* 163 detected in book pages; and day-to-day experimental replicates of book page sampling (Figures S1–S18); additional references (PDF)
