# Supplementary material for: Unifying Passive Air Sampling with Paper Spray Ionization Mass Spectrometry for the Detection of Third-Hand Cigarette Smoke
Source: J Am Soc Mass Spectrom. 2026 Sep 8;37(10):2534–41. doi: 10.1021/jasms.6c00271 (PMC13651622; doi:10.1021/jasms.6c00271)
Supplement: Supplementary file 1 [file js6c00271_si_001.pdf]

SUPPORTING INFORMATION

Unifying Passive Air Sampling with Paper Spray Ionization Mass Spectrometry for the Detection of Third-Hand Cigarette Smoke

Bo-Cheng Ke, Noor Hidayat Abu Bakar, Pawel L. Urban*

Department of Chemistry, National Tsing Hua University

101, Section 2, Kuang-Fu Rd., Hsinchu, 300044, Taiwan

* Corresponding author:

P.L. Urban (urban@mx.nthu.edu.tw)

ADDITIONAL FIGURES

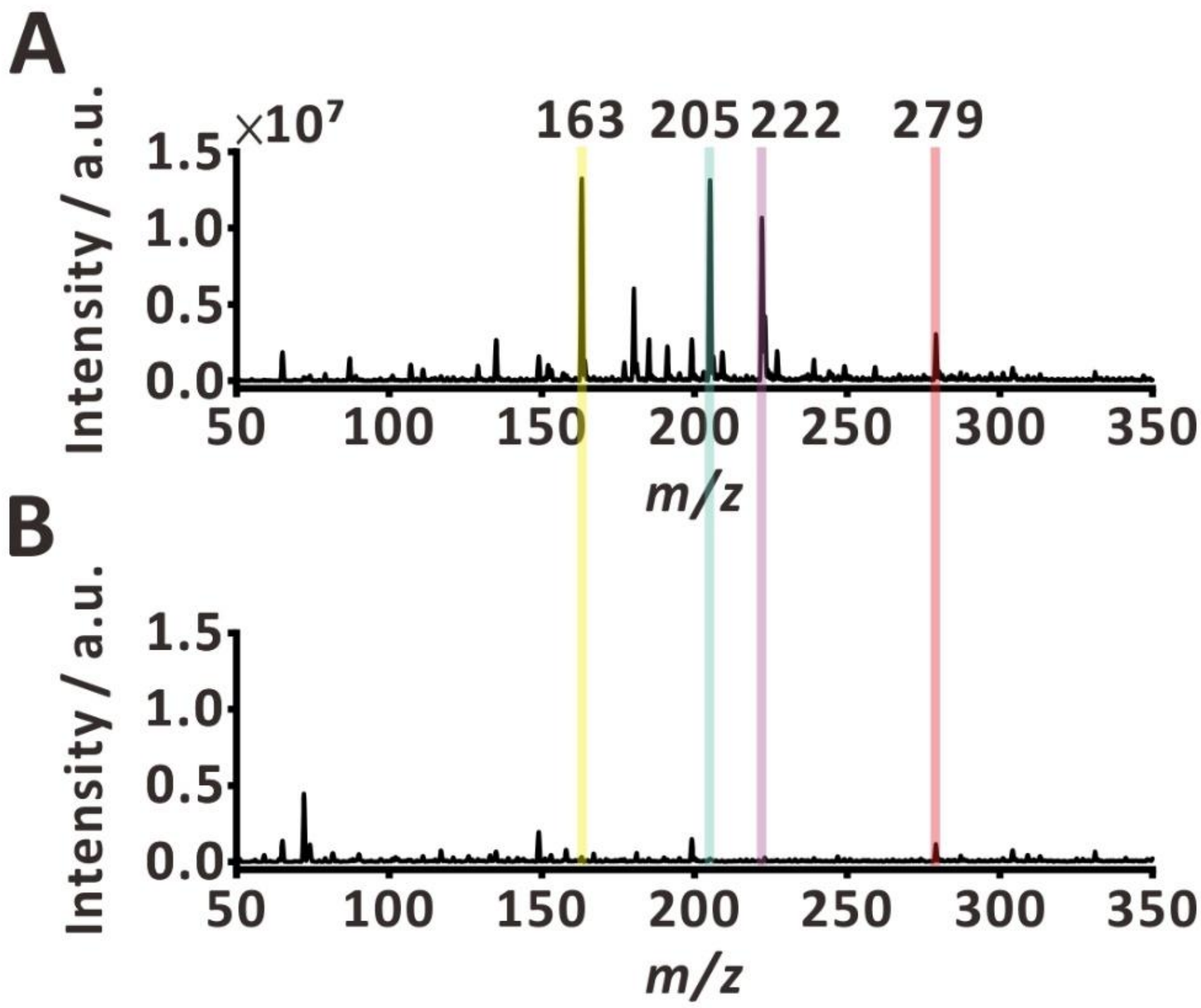

Figure S1. Comparison of PSI mass spectra obtained using uncleaned (A) and cleaned (B) paper substrate.

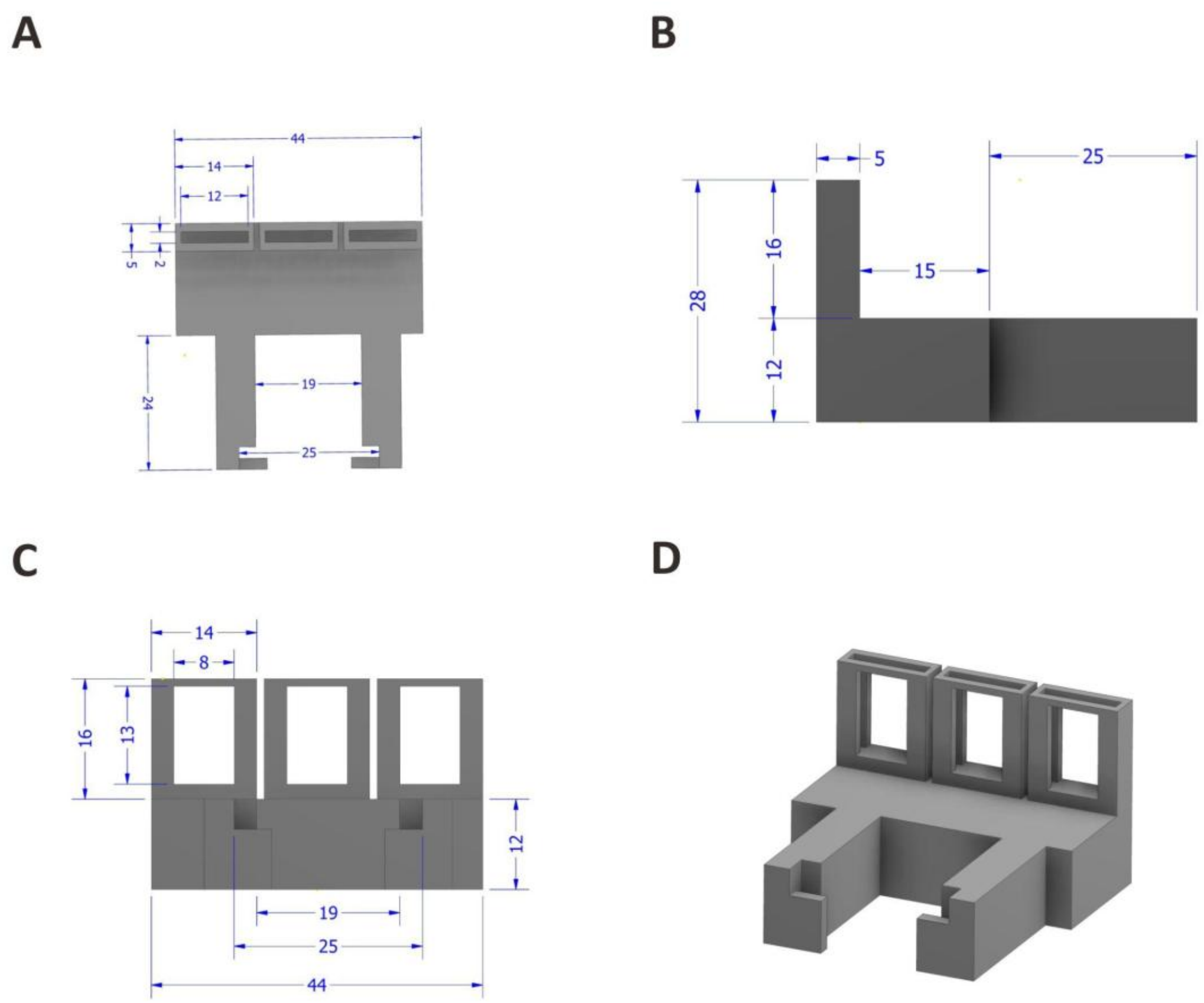

Figure S2. Technical drawing of the passive sampler holder: (A) top view; (B) side view; (C) front view; (D) three-dimensional view. Units within the drawing: mm.

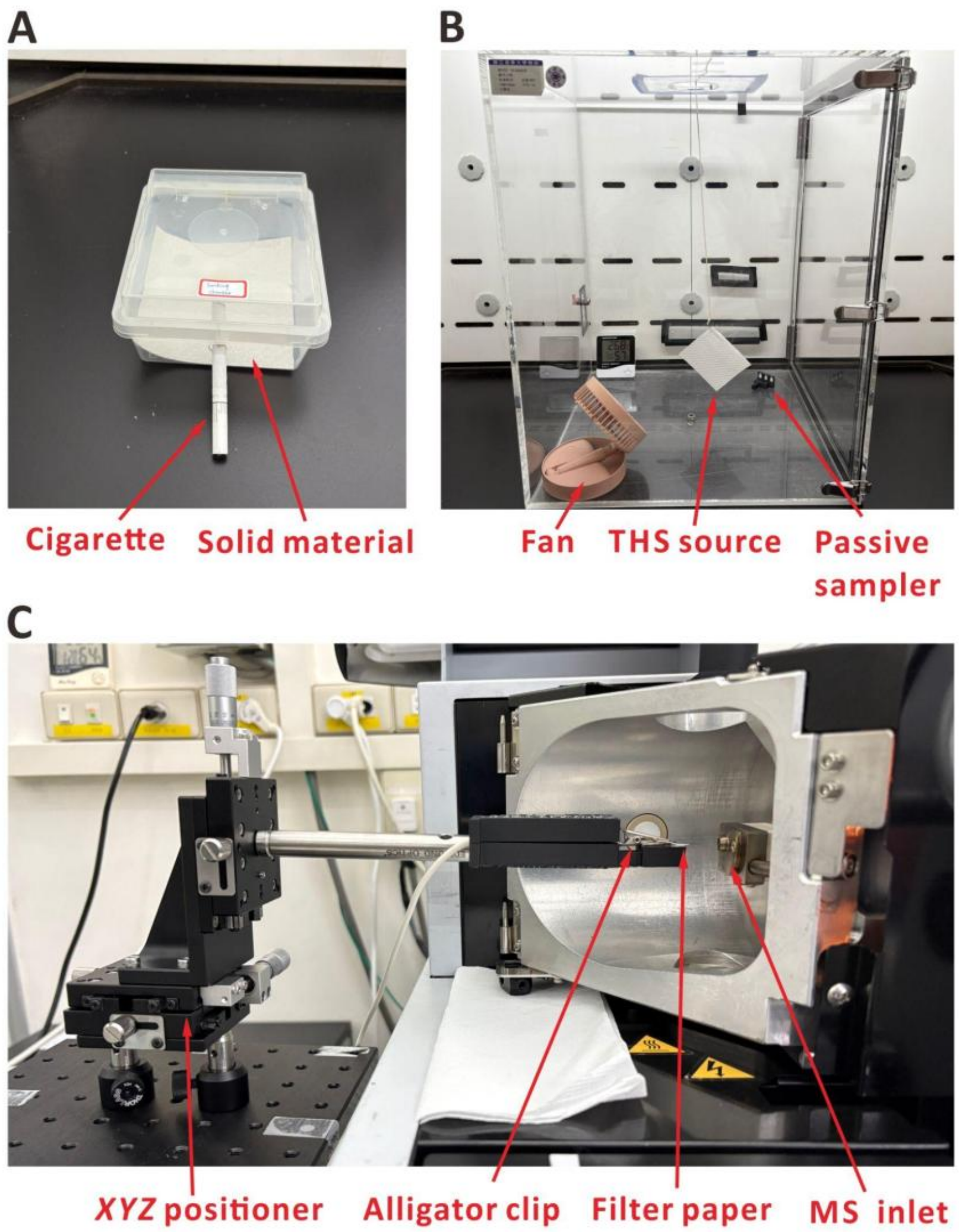

Figure S3. Photos of the experimental setup used in this study: (A) generation of SHS; (B) exposure of passive sampler to THS; (C) PSI-MS analysis of the paper probe.

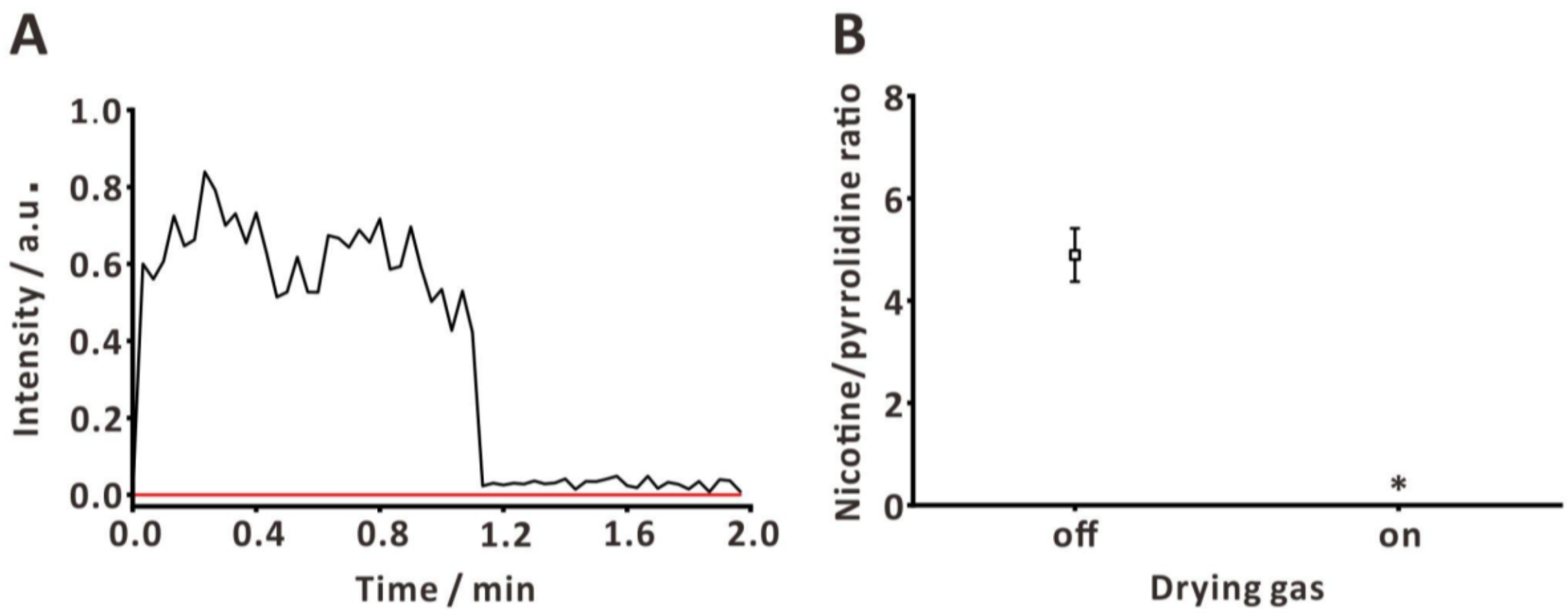

Figure S4. Influence of drying gas. (A) Extracted ion current (EIC) of nicotine (*m/z* 163) under different drying gas conditions. The red line represents the EIC with the drying gas turned on, while the black line corresponds to the EIC without the drying gas. (B) Comparison of the nicotine-to-pyrrolidine signal ratio under drying gas on and off conditions. The asterisk in (B) "*" indicates that pyrrolidine and nicotine were not detected when the drying gas was switched on.

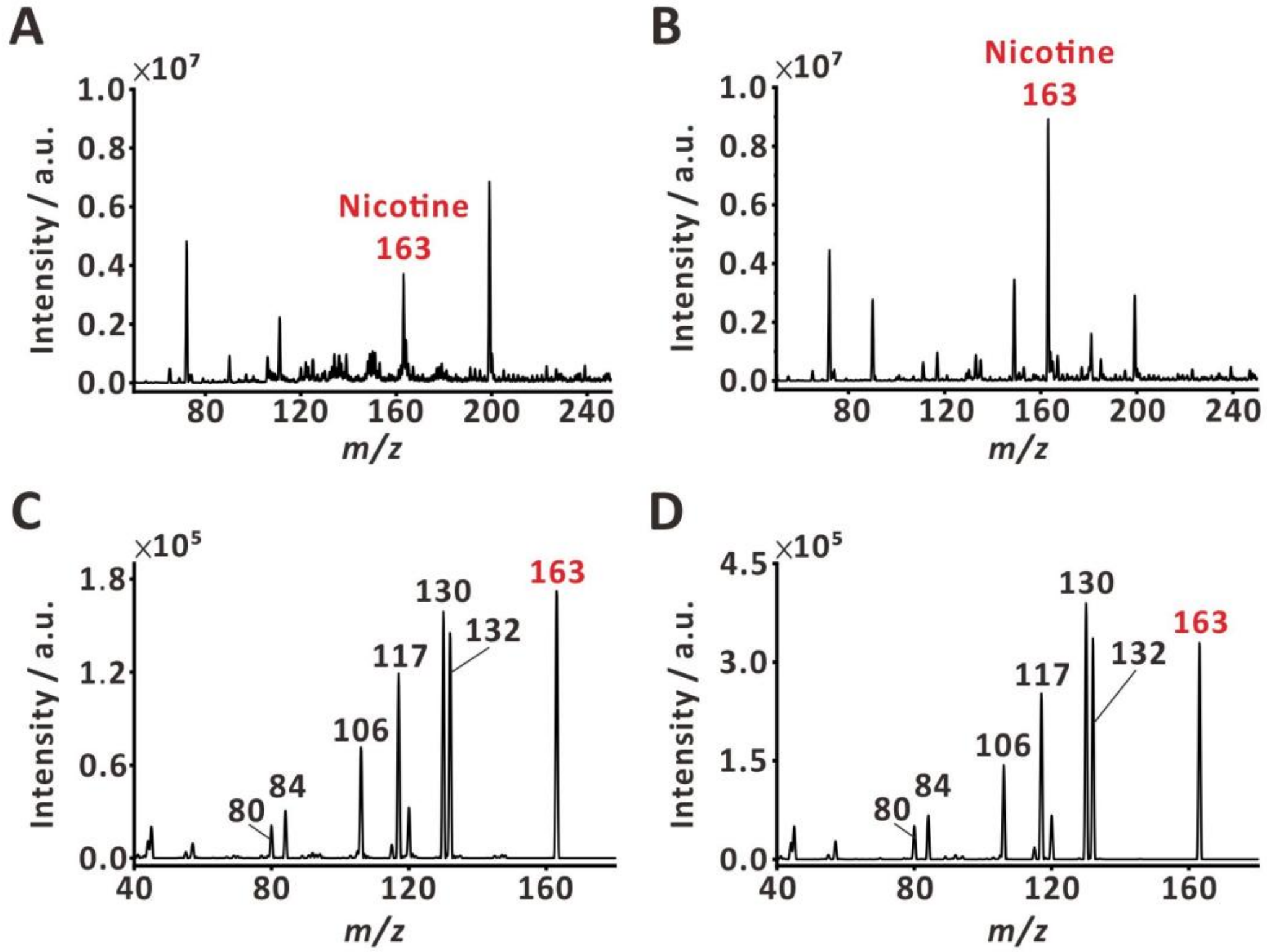

Figure S5. PSI-MS analysis of THS and nicotine standard: mass spectrum of THS (A) and nicotine standard (B); tandem mass spectrum of protonated nicotine (precursor ion: *m/z* 163) obtained from THS (C) and protonated nicotine (precursor ion: *m/z* 163) obtained from authentic standard (D).

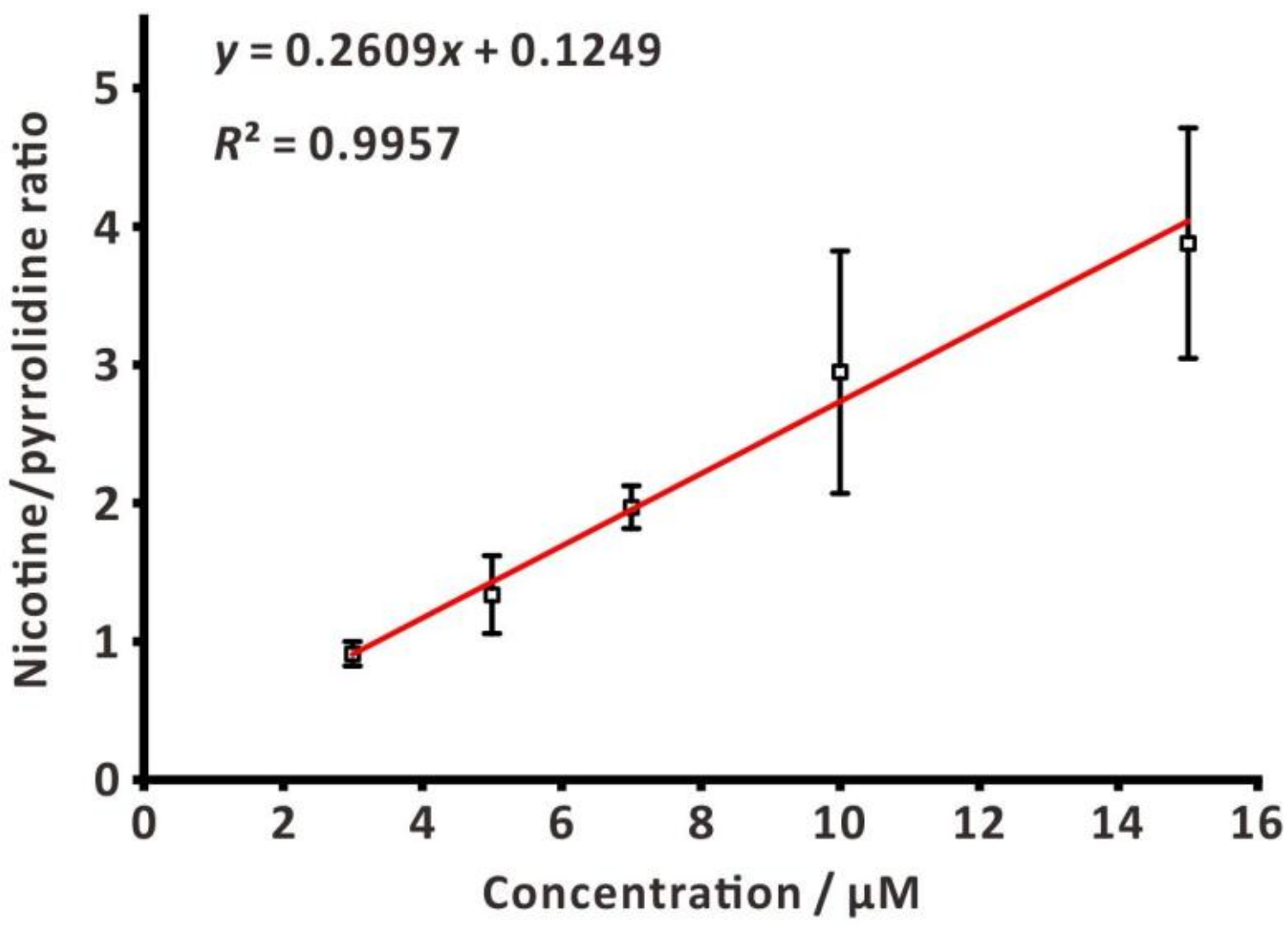

Figure S6. Calibration curve of nicotine (3–15 µM) obtained by PSI-MS. The nicotine-to-pyrrolidine ratio was determined from mass spectra averaged over 0.1–0.6 min by normalizing the nicotine signal against the pyrrolidine signal. The red line indicates the linear regression fit. Error bars represent mean ± standard deviation ($n = 3$) at each concentration.

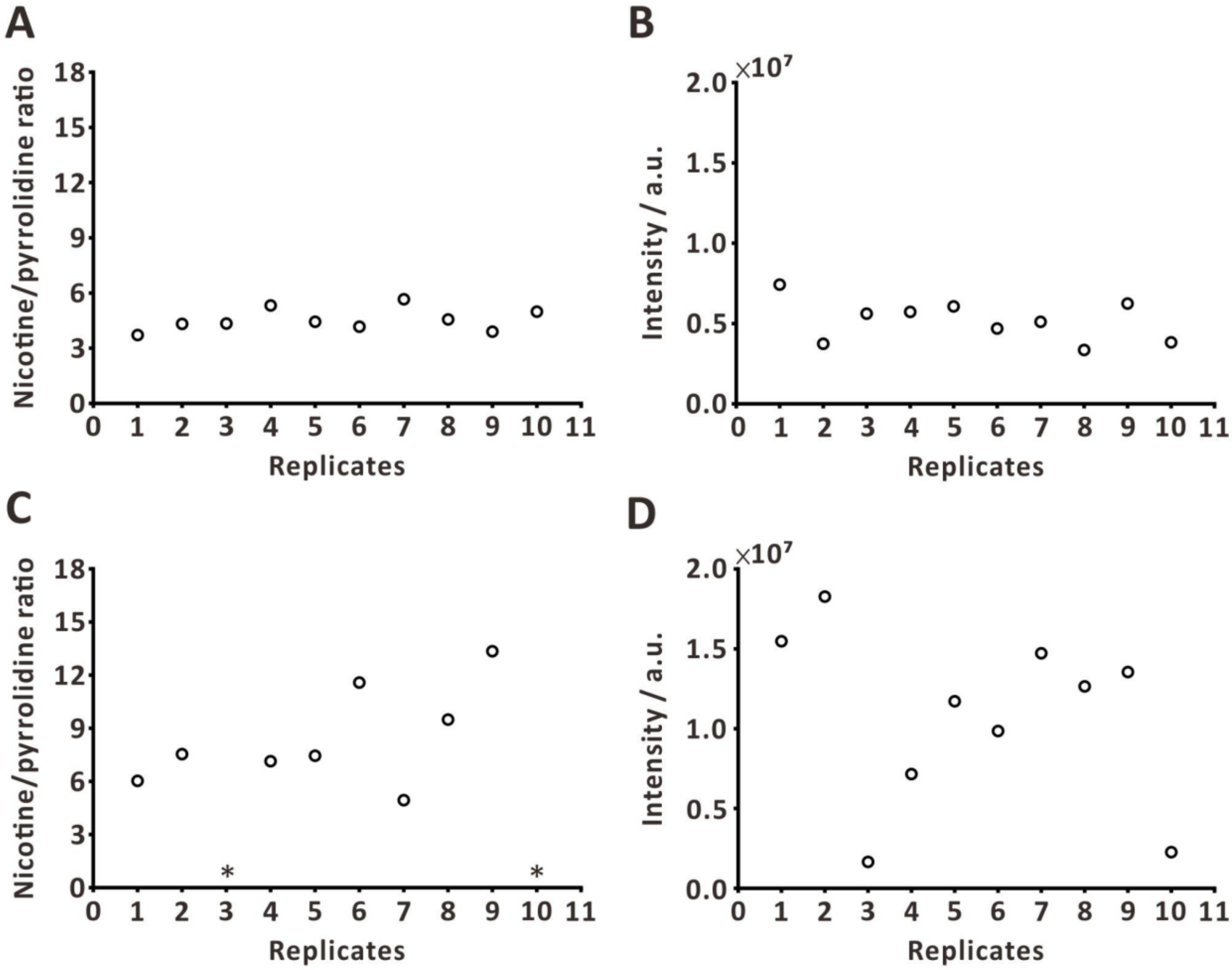

Figure S7. Repeatability test for (A) 10 μM nicotine standard expressed as the nicotine-to-pyrrolidine signal ratio (*m/z* 163 for nicotine and *m/z* 72 for pyrrolidine); (B) 10 μM nicotine standard expressed as signal intensity of nicotine (*m/z* 163); (C) nicotine from THS expressed as the nicotine-to-pyrrolidine signal ratio; and (D) nicotine from THS expressed as signal intensity of nicotine (*m/z* 163). The asterisk in (C) "*" indicates that pyrrolidine was not detected in replicates 3 and 10.

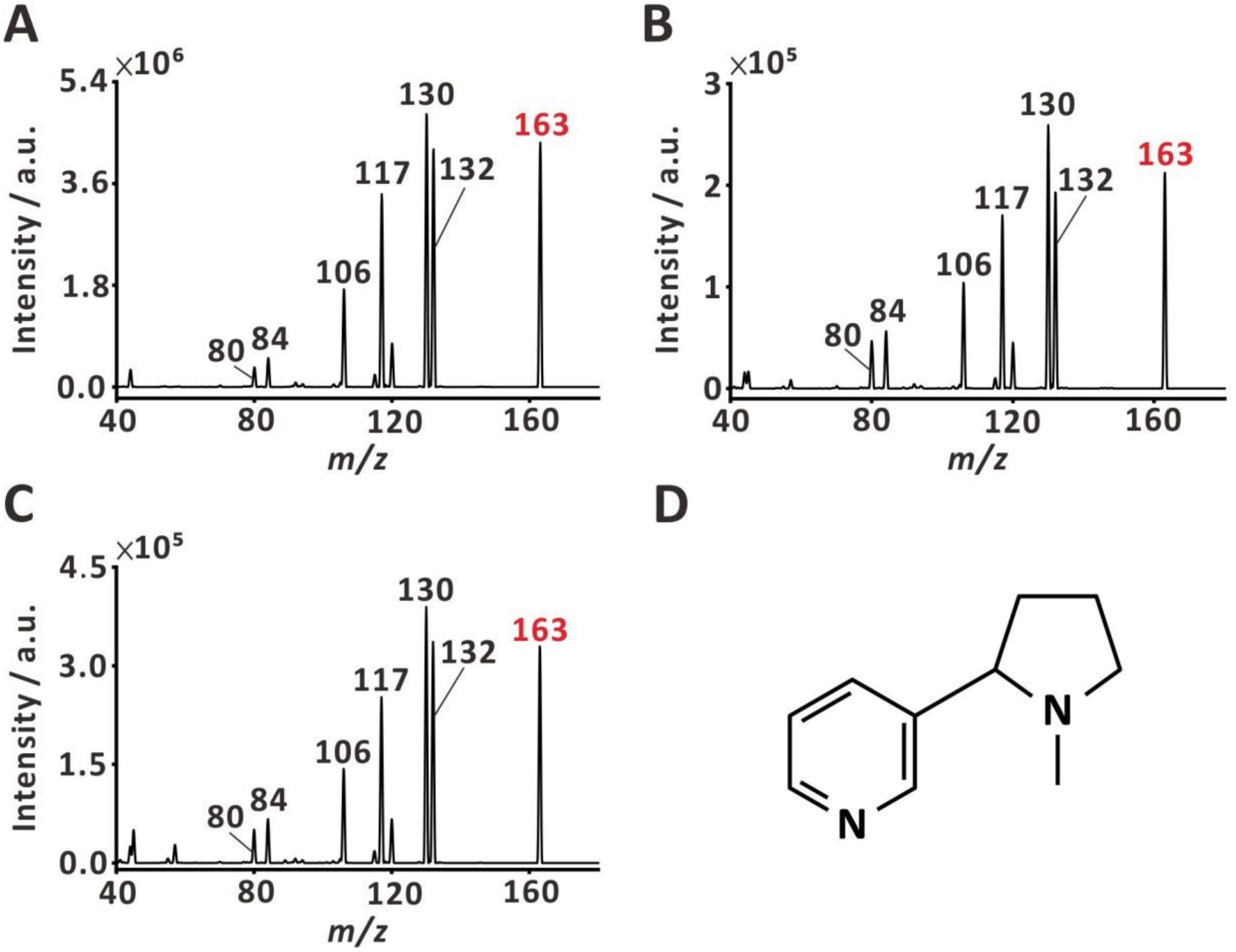

Figure S8. Tandem mass spectra of nicotine (*m/z* 163) obtained from (A) SHS, (B) THS, and (C) authentic standard. (D) Structure of nicotine.

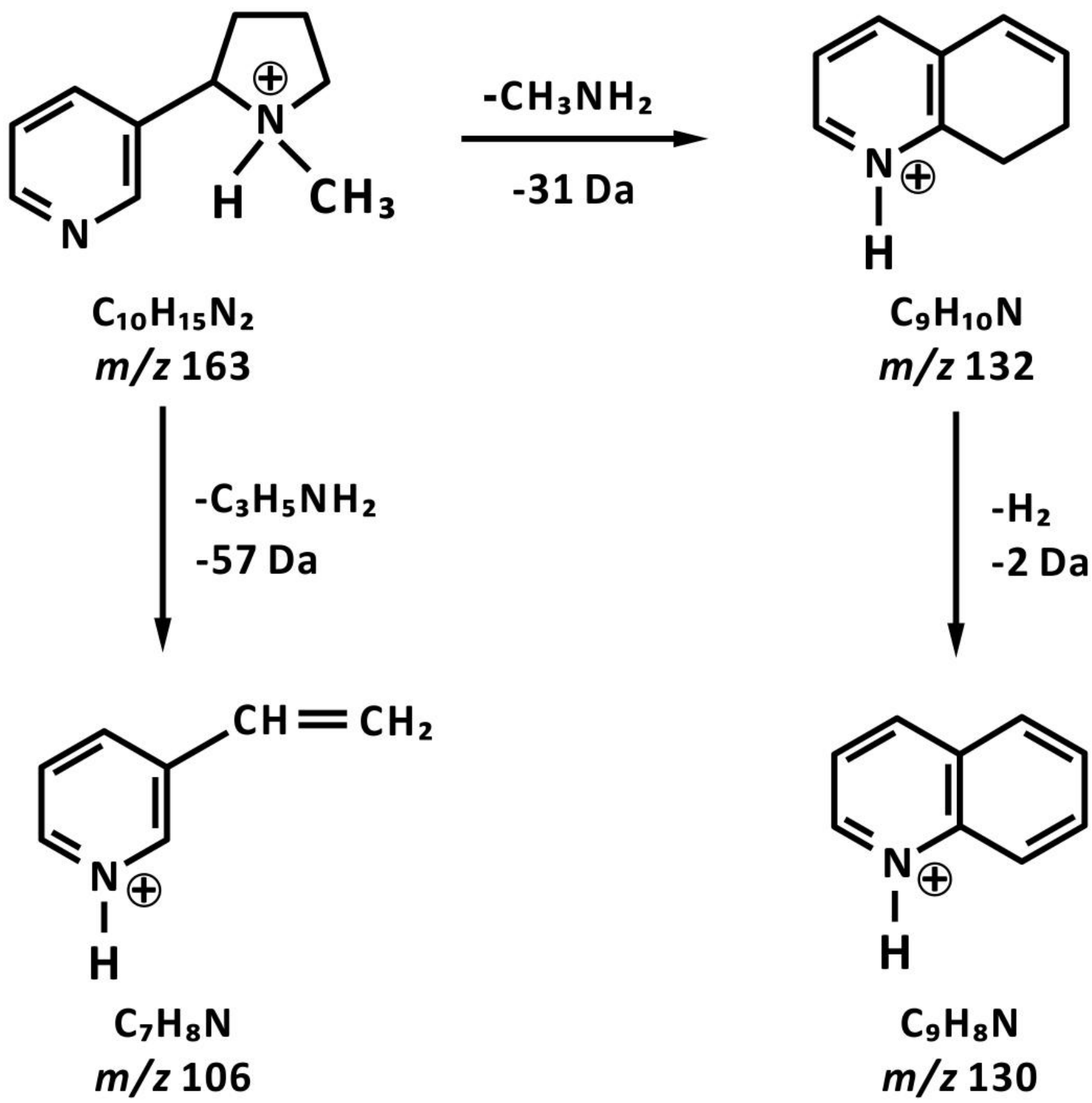

Figure S9. Suggested fragmentation pathway of protonated nicotine (*m/z* 163). Based on ref.[1]

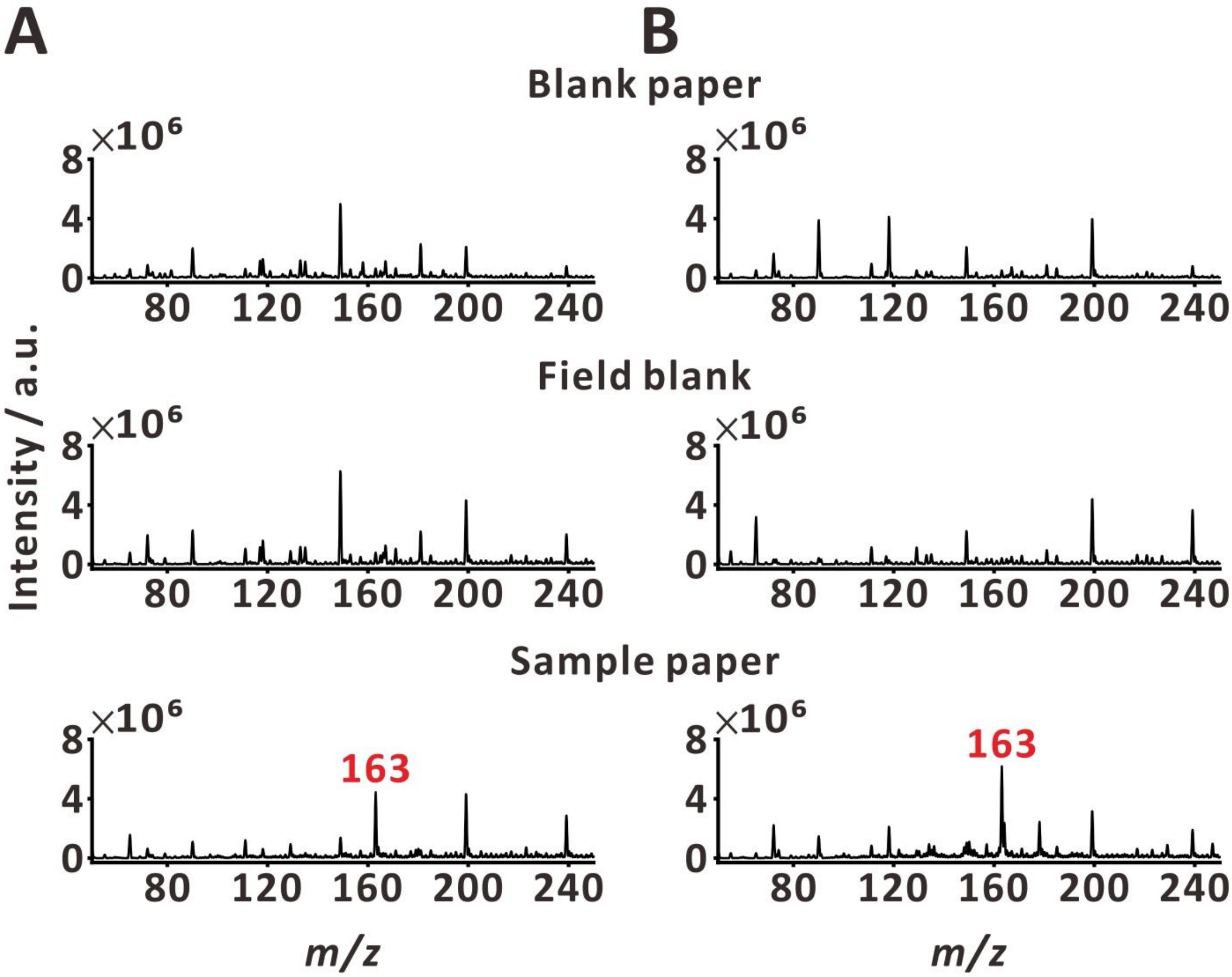

Figure S10. PSI-MS analysis of THS emitted from different materials: (A) cotton cloth, and (B) towel paper (*RH*: 64-72%; *T*: 22.4-23.0°C).

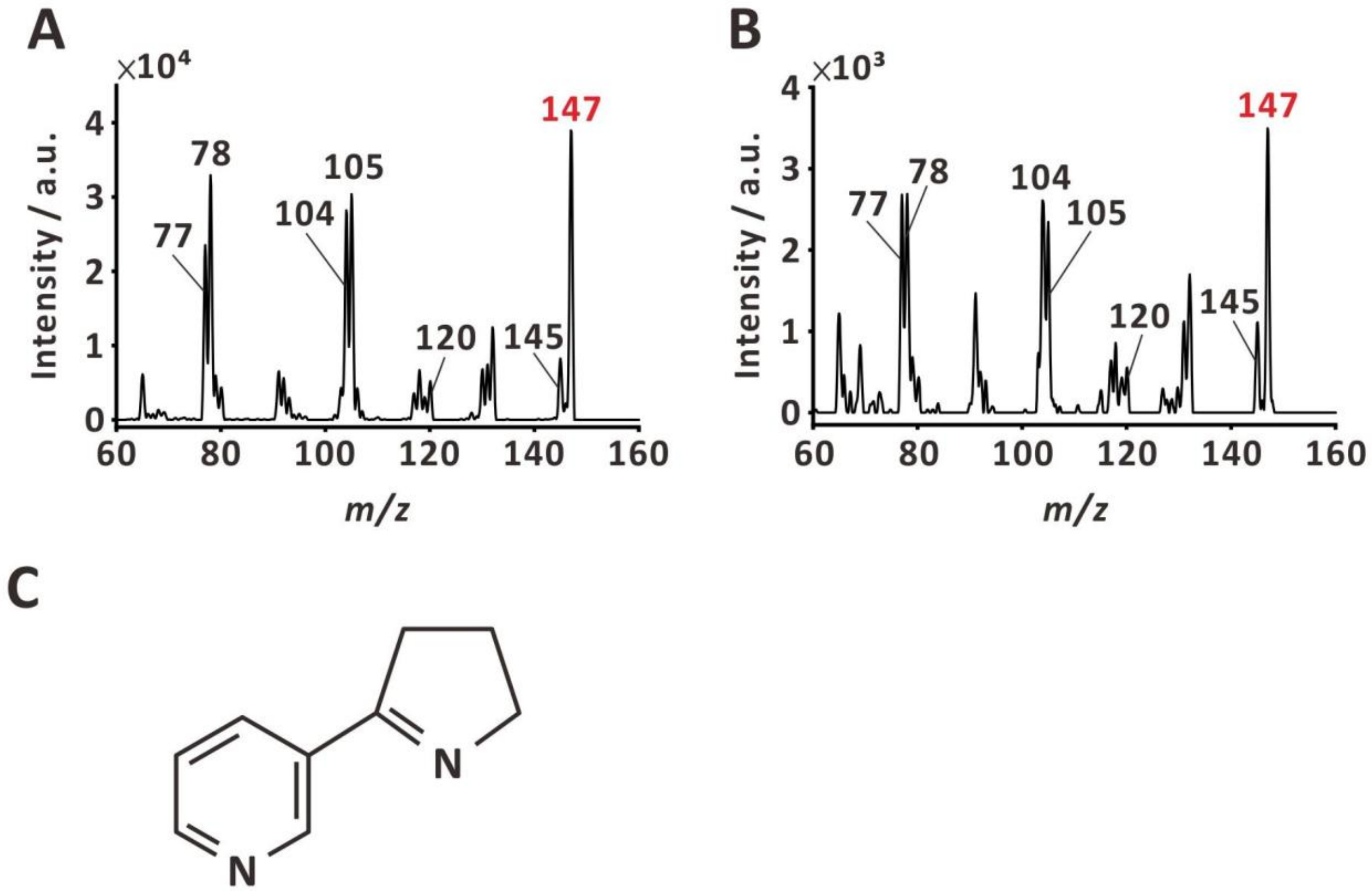

Figure S11. Tandem mass spectra of myosmine (*m/z* 147) obtained from (A) SHS and (B) THS. (C) Structure of myosmine.

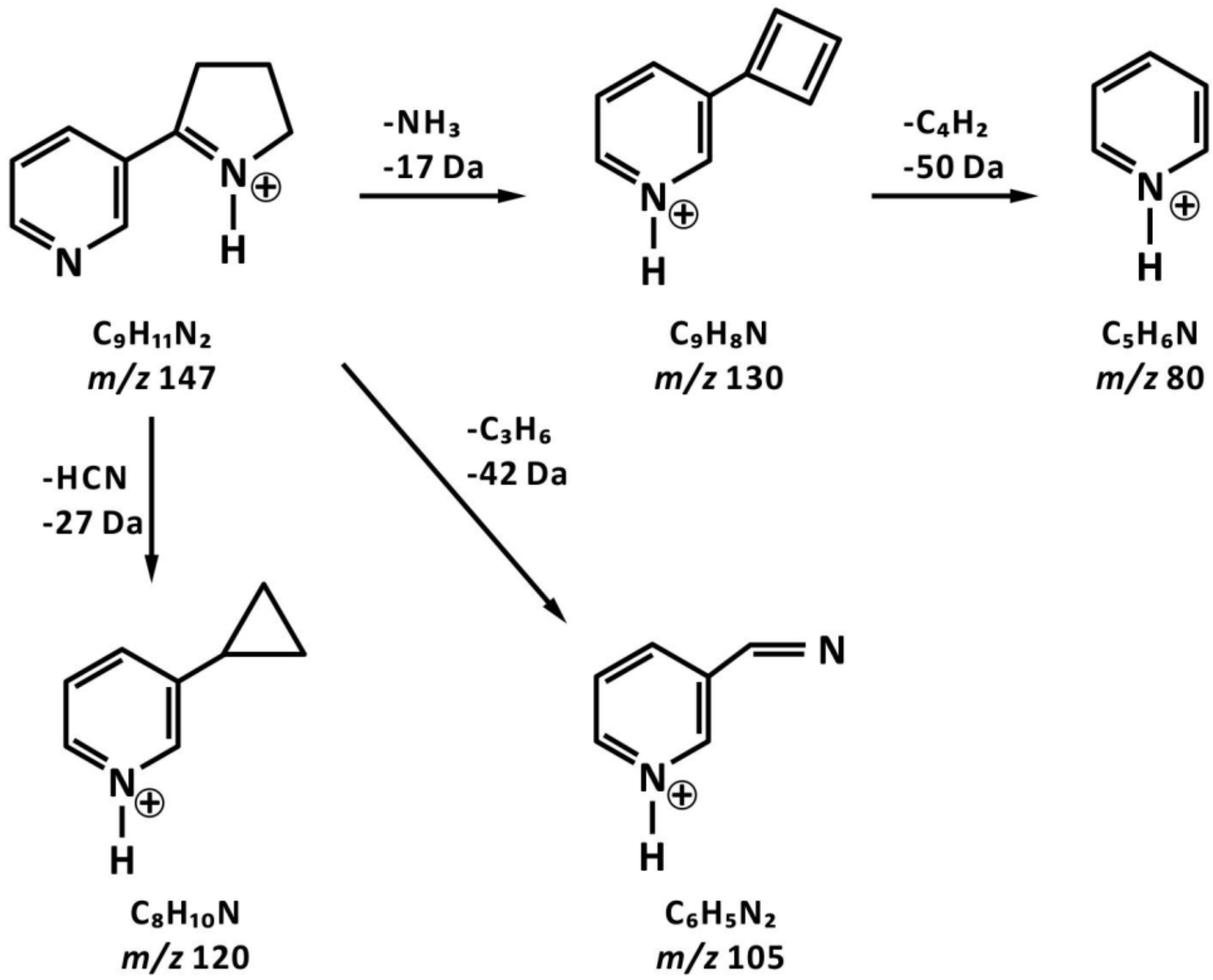

Figure S12. Suggested fragmentation pathway of protonated myosmine (*m/z* 147). Based on ref.[2]

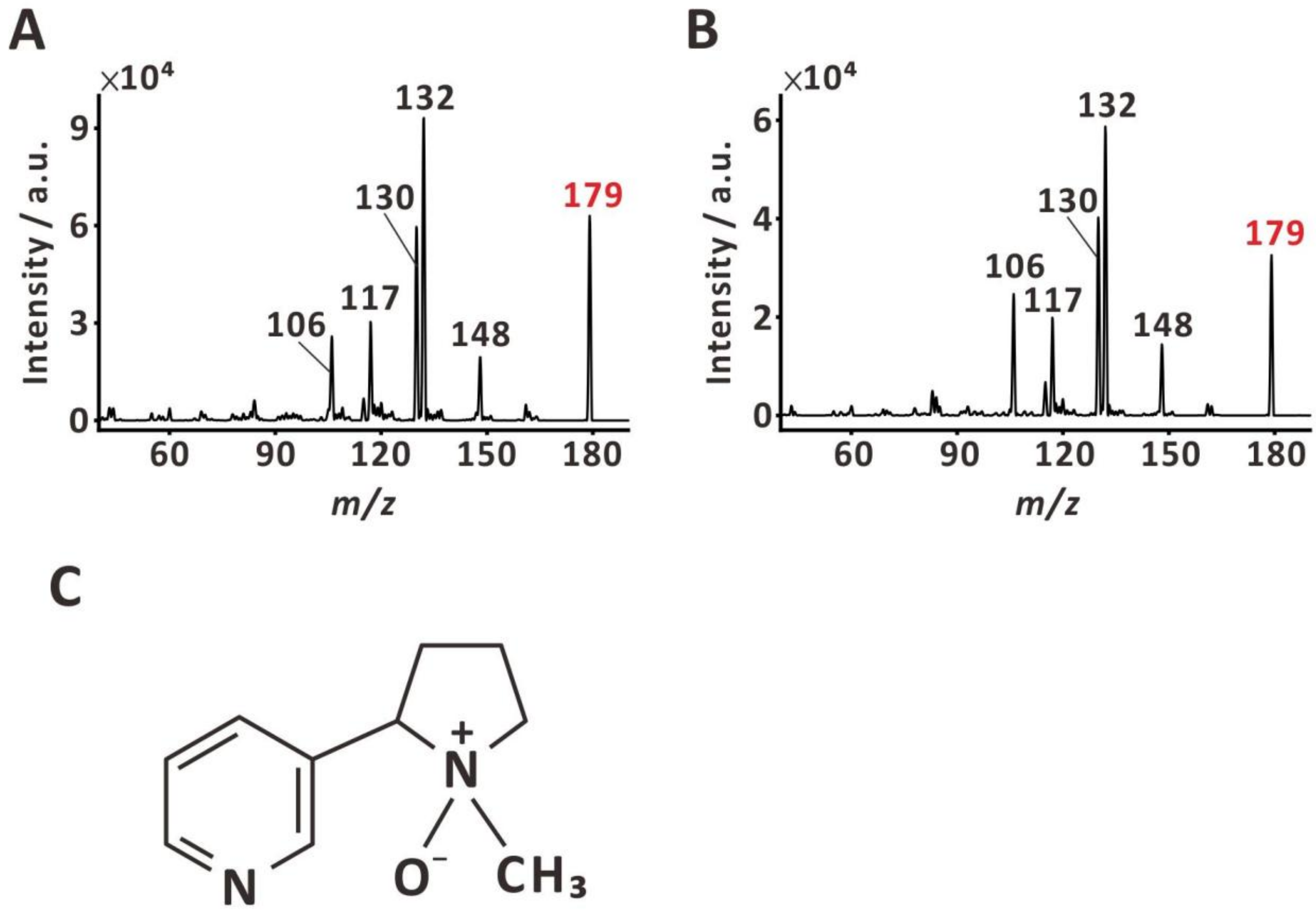

Figure S13. Tandem mass spectra of nicotine 1′-*N*-oxide (*m/z* 179) obtained from (A) SHS, (B) THS. (C) Structure of nicotine 1′-*N*-oxide.

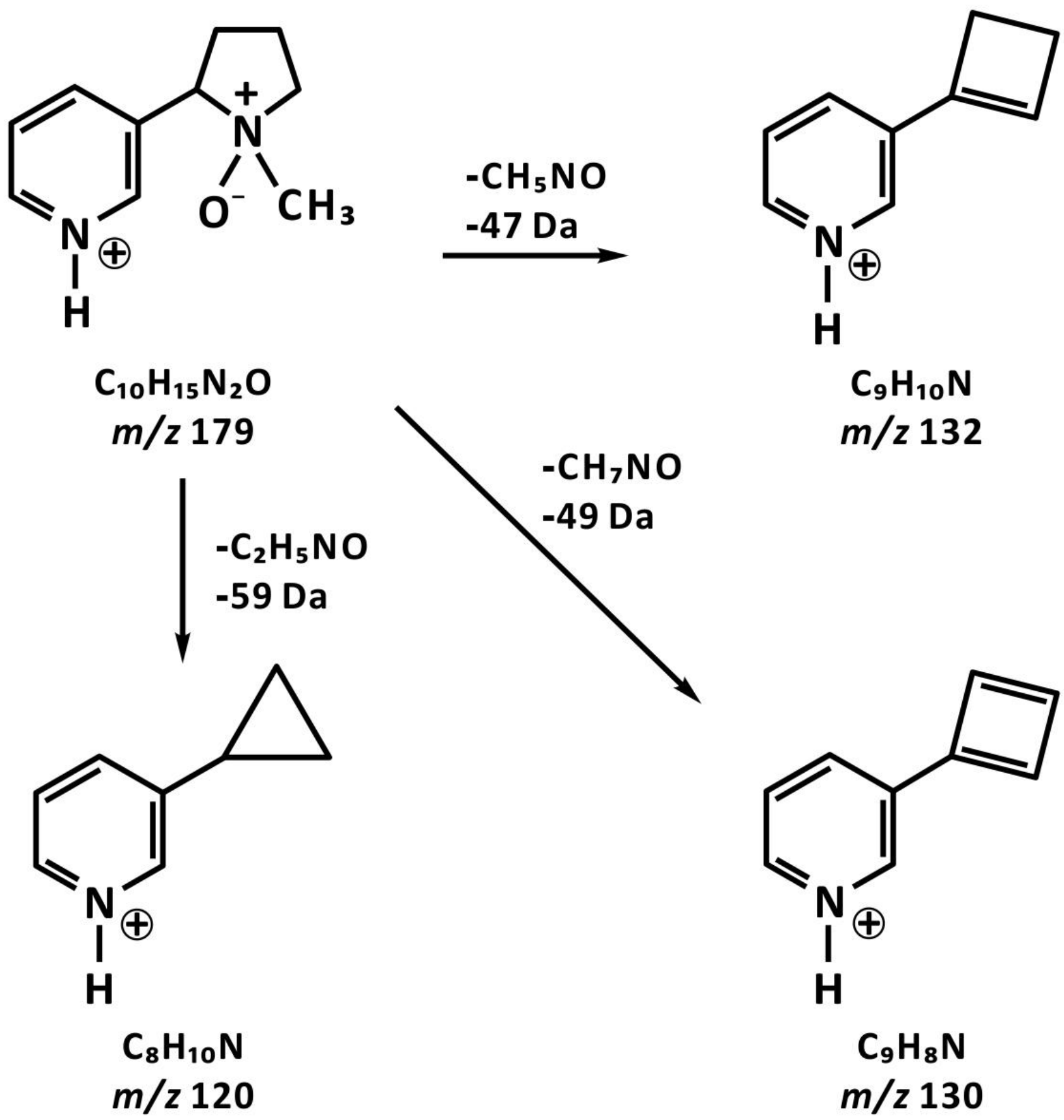

Figure S14. Suggested fragmentation pathway of protonated nicotine 1′-*N*-oxide (*m/z* 179). Based on refs.[3,4]

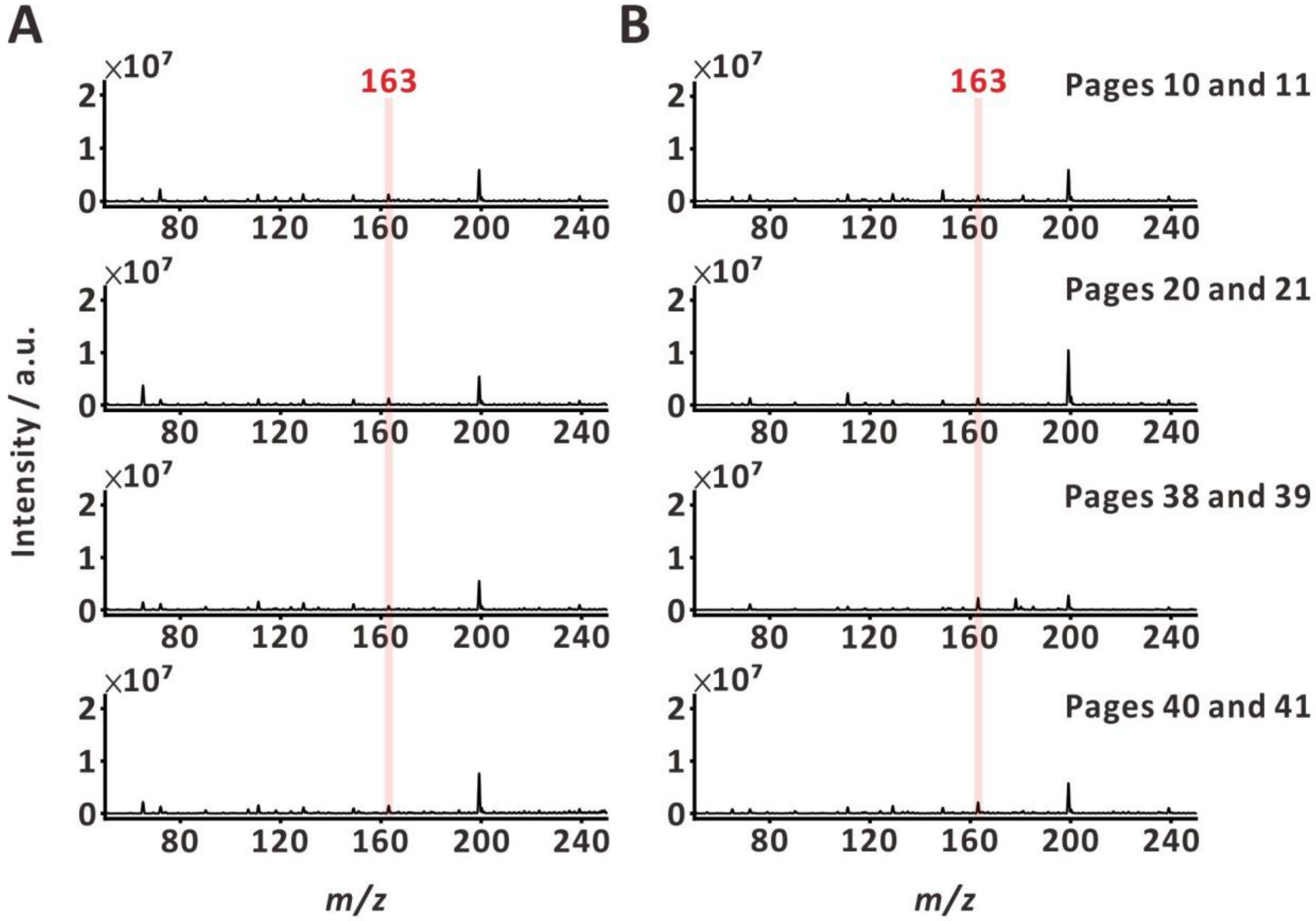

Figure S15. Mass spectra obtained from book pages (10, 11, 20, 21, 38, 39, 40, and 41): (A) field blank analysis before the tobacco smoke exposure; and (B) second sampling performed 1 h after the initial measurement following smoke exposure.

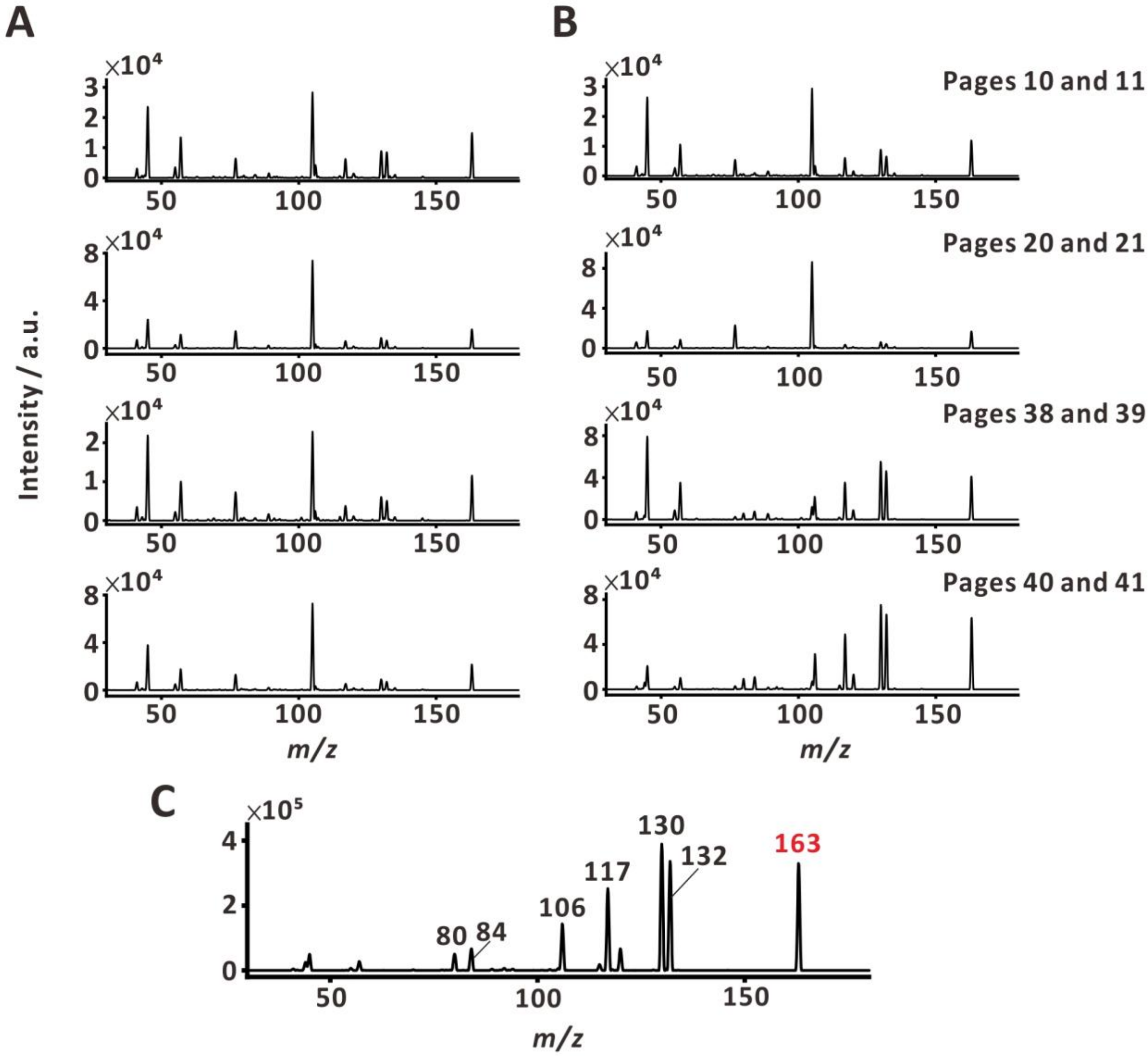

Figure S16. Tandem mass spectra of precursor ion *m/z* 163: (A) field blank analysis of book pages before the tobacco smoke exposure; (B) second sampling performed 1 h after the initial measurement following smoke exposure; (C) authentic standard of 10 μM nicotine.

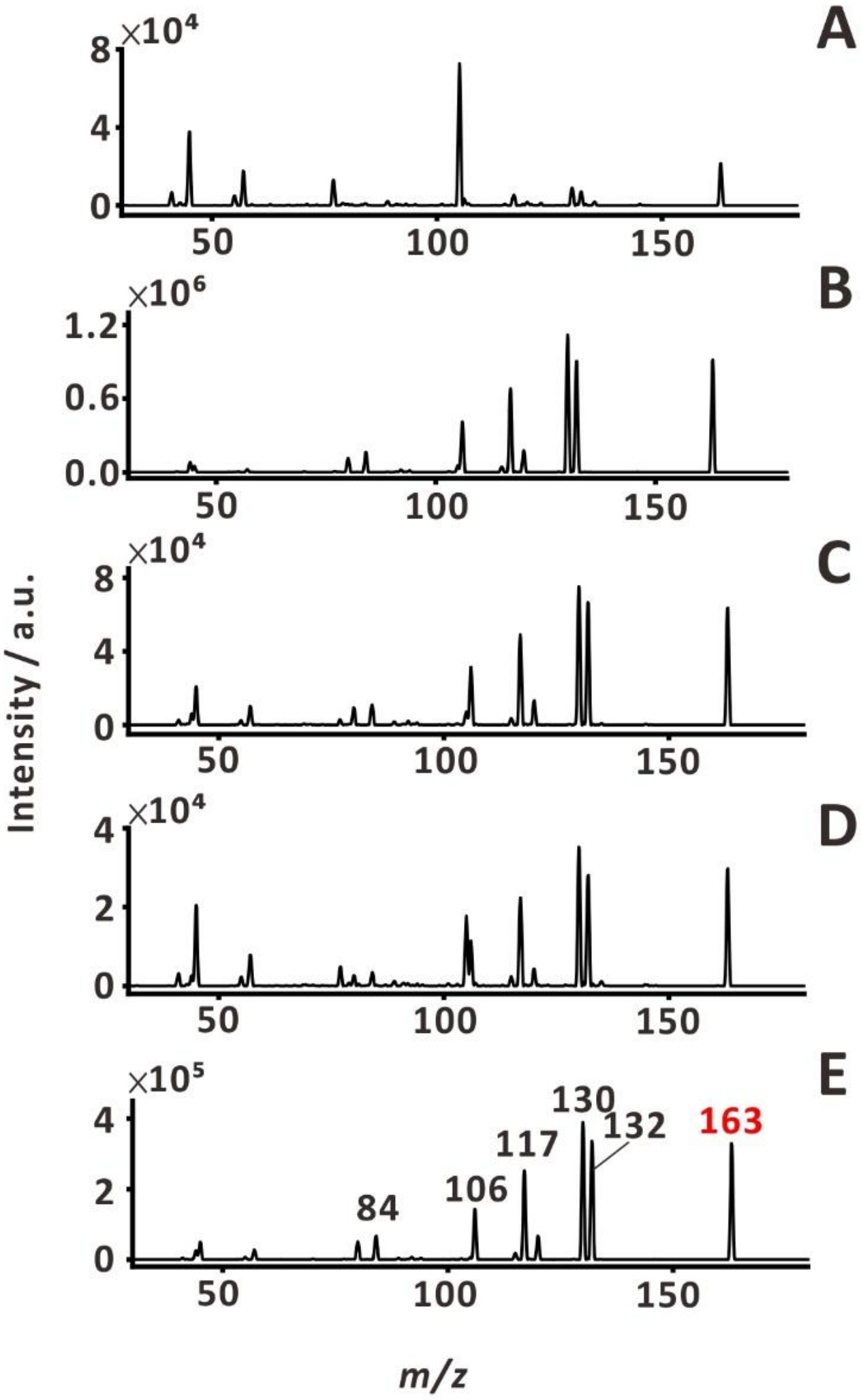

Figure S17. Tandem mass spectra of precursor at *m/z* 163 detected in exposed pages (40 and 41): (A) field blank; (B) first sampling immediately after exposure; (C) second sampling 1 h after initial measurement; (D) third sampling 4 days after initial measurement; (E) authentic standard of nicotine (10 μM).

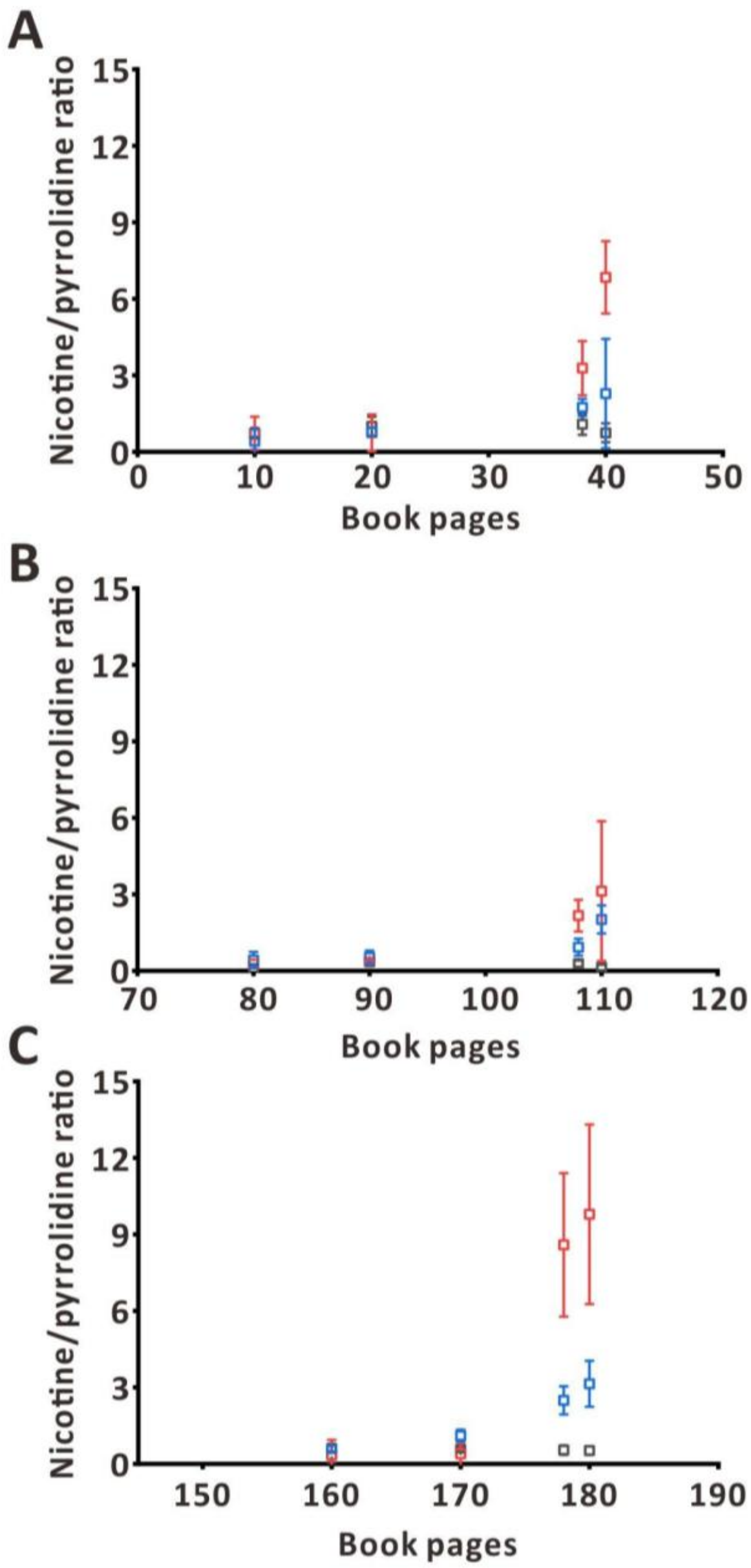

Figure S18. Day-to-day experimental replicates of nicotine-to-pyrrolidine ratios measured on different book pages following exposure to tobacco smoke (pages 40, 110, and 180). Panels A–C correspond to independent measurements conducted on separate days (April 10th, 14th, and 23rd, 2026). Error bars represent standard deviation (n = 3). Gray symbols represent the field blank analysis before tobacco smoke exposure; red symbols represent the initial sampling performed immediately after tobacco smoke exposure; blue symbols represent the second sampling performed 1 h after the initial sampling.

ADDITIONAL REFERENCES

(1) Smyth, T. J.; Ramachandran, V. N.; McGuigan, A.; Hopps, J.; Smyth, W. F. Characterisation of Nicotine and Related Compounds Using Electrospray Ionisation with Ion Trap Mass Spectrometry and with Quadrupole Time-of-Flight Mass Spectrometry and Their Detection by Liquid Chromatography/Electrospray Ionisation Mass Spectrometry. *Rapid Commun. Mass Spectrom.* **2007**, *21*, 557–566.

(2) Medana, C.; Baiocchi, C.; Dal Bello, F.; Aigotti, R.; Sala, C.; Santoro, V.; Gastaldi, D. Analysis of Nicotine Alkaloids and Impurities in Liquids for e-Cigarettes by LC–MS, GC–MS, and ICP-MS. *Curr. Trends Mass Spectrom.* **2016**, *14*, 20–28.

(3) Medana, C.; Santoro, V.; Bello, F. D.; Sala, C.; Pazzi, M.; Sarro, M.; Calza, P. Mass Spectrometric Fragmentation and Photocatalytic Transformation of Nicotine and Cotinine: MS and Photocatalysis of Nicotine and Cotinine. Rapid Commun. Mass Spectrom. **2016**, *30*, 2617–2627.

(4) Uhlig, S.; Olderbø, B. P.; Samuelsen, J. T.; Uvsløkk, S.; Ivanova, L.; Vanderstraeten, C.; Grutle, L. A.; Rangel-Huerta, O. D. Mass Spectrometry-Based Metabolomics Study of Nicotine Exposure in THP-1 Monocytes. *Sci. Rep.* **2024**, *14*, 14957.
